# Oral whole-leaf matcha partially attenuates UV-induced dermoepidermal disruption and collagen phenotype alterations in a rat model of repeated photoaging

**DOI:** 10.3389/fmed.2026.1813454

**Published:** 2026-06-10

**Authors:** Özge Zorlu, Sevil Karabağ, Mazhar Özkan, Melisa Beyhan Yılmaz, Seydi Yıkmış

**Affiliations:** 1 Department of Dermatology and Venereology, Faculty of Medicine, Tekirdağ Namık Kemal University, Tekirdağ, Türkiye; 2 Department of Pathology, Faculty of Medicine, Tekirdağ Namık Kemal University, Tekirdağ, Türkiye; 3 Department of Anatomy, Faculty of Medicine, Tekirdağ Namık Kemal University, Tekirdağ, Türkiye; 4 Department of Tumor Biology and Immunology, Health Sciences Institute, Tekirdağ Namık Kemal University, Tekirdağ, Türkiye; 5 Department of Food Technology, Tekirdağ Namık Kemal University, Tekirdağ, Türkiye

**Keywords:** collagen phenotype, dermoepidermal junction, matcha, oxidative stress, photoaging, UV radiation

## Abstract

**Background:**

Repeated ultraviolet (UV) exposure promotes oxidative stress, extracellular matrix remodeling, and disruption of dermoepidermal architecture, all of which contribute to skin photoaging. Although green tea derivatives have been investigated for photoprotective effects, the impact of oral whole-leaf matcha powder on cumulative UV-induced skin changes remains insufficiently characterized. The aim of this study was to investigate whether oral whole-leaf matcha powder was associated with attenuation of UV-induced dermoepidermal disruption, collagen phenotype alterations, and skin surface changes in a rat model of repeated UV exposure.

**Methods:**

Thirty-two female Sprague–Dawley rats were randomly assigned to Control, UV, Matcha, and UV + Matcha groups (*n* = 8/group). Matcha was administered orally at 50 mg/kg/day for 30 days. Experimental photoaging was induced using a repeated UV exposure protocol comprising 15 sessions over 4 weeks. Histomorphometric evaluation of dermoepidermal architecture, collagen phenotype, and elastic fiber parameters was performed together with serum antioxidant enzyme measurements and macroscopic skin surface analyses.

**Results:**

Compared with controls, repeated UV exposure increased epidermal thickness (*p* = 0.001), reduced the interdigitation index (*p* = 0.002), decreased type I collagen and the type I/III collagen ratio (*p* < 0.001 and *p* = 0.001, respectively), increased type III collagen (*p* = 0.016), and reduced glutathione reductase activity (*p* < 0.001). UV exposure was also associated with higher surface texture indices, including FFT roughness and local variance (both *p* < 0.001), as well as an increased fine wrinkle percentage (*p* < 0.001). Compared with the UV group, the UV + Matcha group showed attenuated epidermal thickening (*p* = 0.029), higher dermal papilla thickness (*p* = 0.036) and epidermal rete ridge thickness (*p* < 0.001), higher superoxide dismutase activity (*p* = 0.031), a lower local variance index (*p* = 0.037), and reduced fine wrinkle percentage (*p* = 0.001).

**Conclusion:**

Oral whole-leaf matcha powder was associated with partial attenuation of UV-induced phenotypic changes, including dermoepidermal structural alterations, collagen phenotype shifts, and surface deterioration, in this rat model of repeated photoaging. These findings suggest a potential adjunctive photoprotective effect, although further molecular-level validation and clinical studies are required to clarify the underlying pathways and translational relevance.

## 1 Introduction

Ultraviolet (UV) radiation is a major extrinsic driver of skin aging, commonly referred to as photoaging. UVA (λ = 320–400 nm) penetrates more deeply into the dermis and predominantly promotes oxidative stress through reactive oxygen species (ROS), lipid and protein oxidation, activation of activator protein-1 (AP-1) and mitogen-activated protein kinase (MAPK) signaling, upregulation of matrix metalloproteinases (MMPs), and suppression of TGF-β-dependent collagen homeostasis; collectively, these events accelerate collagen degradation and extracellular matrix disorganization. In contrast, UVB (λ = 290–320 nm) acts more strongly on the epidermis and contributes to direct DNA damage, inflammatory signaling, keratinocyte stress responses, and oxidative injury. Repeated UV exposure, therefore, creates a self-amplifying cycle of DNA damage, ROS production, inflammation, and cellular senescence, which further propagates matrix remodeling and tissue injury. At the tissue level, these processes are accompanied by keratinocyte hyperproliferation and abnormal epidermal remodeling, leading to epidermal thickening. Concomitantly, collagen fragmentation, altered collagen phenotype, elastic fiber degeneration, and solar elastosis contribute to wrinkle formation, loss of elasticity, and progressive disruption of dermoepidermal architecture (1–3).

Matcha is a finely powdered form of Japanese green tea derived from the shade-grown young leaves of *Camellia sinensis* (L) Kuntze, which are steamed, dried, deveined, and stone-ground after harvesting, thereby allowing consumption of the entire leaf. This cultivation and processing method results in relatively higher levels of certain bioactive compounds compared to conventional green tea infusions. Matcha powder contains both water-insoluble (e.g., fat-soluble vitamins, chlorophylls, dietary fibers, and proteins) and water-soluble (e.g., polyphenols, caffeine, water-soluble vitamins, dietary fibers, and amino acids) constituents (4, 5).

Matcha may function differently from traditional loose-leaf green tea owing to its higher levels of polyphenols, catechins, caffeine, theanine, and other bioactive compounds (4, 5). Among these, catechins and related flavonoids, major polyphenolic constituents biosynthetically linked to the plant phenylpropanoid pathway, are of particular interest because they have been widely investigated for their antioxidant, anti-inflammatory, and photoprotective properties, including the ability to counteract UV-induced oxidative stress, inflammatory signaling, and extracellular matrix degradation (4, 6–10). Owing to this compositional profile, matcha has attracted increasing scientific interest for its antioxidant, anti-inflammatory, immunomodulatory, and potential photoprotective properties (4, 11–14). Reported benefits include enhancement of cognitive function, stress reduction, cardiometabolic protection, and potential anti-tumor effects in breast cancer; however, existing human and animal studies are heterogeneous, often limited in size, and predominantly focused on isolated catechins or processed green tea extracts rather than whole-leaf preparations (4, 5, 15, 16).

The chemopreventive benefits of green tea polyphenols against UVB-induced skin carcinogenesis—by mitigating UVB-mediated DNA damage, oxidative stress, and inflammation, preserving endogenous antioxidant enzyme activities, down-regulating MMP expression, and protecting collagen and elastic fibers—have been demonstrated in murine skin models (17). Clinical studies in humans likewise suggest that regular oral supplementation with green tea catechins can reduce erythema response to UV exposure and modulate inflammatory mediators (18). Moreover, green tea and its bioactive constituents have also been incorporated into commercially available sun-protective and cosmetic products. However, the regulatory status and safety expectations of plant-derived ingredients depend on product category, intended use, and labeling claims.

To date, the effects of whole-leaf matcha powder or its complete spectrum of constituents on UV-induced structural remodeling in experimental photoaging models remain insufficiently characterized. Most investigations rely on processed green tea extracts or isolated polyphenols, short-term UV exposure, and limited structural and functional endpoints. This gap leaves it unclear whether the distinct phytochemical profile of matcha confers differential protection against photoaging compared with standard green tea extracts. Additionally, questions remain regarding optimal dosages, the time course of protective effects, and whether matcha’s effects on photoaging-related skin changes, histopathological findings, and molecular markers of skin aging have been robustly demonstrated.

Despite increasing public awareness of photoprotection, excessive sun exposure remains common, highlighting the need for complementary preventive approaches. Given the existing knowledge gap regarding the effects of oral whole-leaf matcha on photoaging, a more rigorous evaluation using a controlled UV-exposure model is warranted. Based on its rich phytochemical composition, we hypothesized that oral whole-leaf matcha powder may attenuate UV-induced structural alterations in the skin. To test this hypothesis, we employed a repeated UV exposure rat model and evaluated dermoepidermal microarchitecture, dermal collagen and elastin integrity, and surface-related parameters. In parallel, serum antioxidant enzyme levels were measured to characterize systemic biochemical responses and explore their potential associations with cutaneous outcomes. Accordingly, we aimed to investigate whether oral whole-leaf matcha powder could attenuate UV-induced changes in dermoepidermal microarchitecture, collagen phenotype, elastic fiber organization, antioxidant enzyme levels, and skin surface characteristics in a rat model of repeated UV exposure.

## 2 Materials and methods

### 2.1 Analysis of phenolic compounds in matcha

#### 2.1.1 Extraction procedure

A defined amount of the tea sample was weighed and transferred into an Erlenmeyer flask. Then, 40 mL of extraction solvent (methanol: chloroform, 70:30, v/v) was added, and the mixture was vortexed for 3 min to ensure homogenization. The sample was subsequently placed in a water bath at 40°C and agitated at 180 rpm for 24 h to complete the extraction. At the end of the extraction period, the mixture was filtered through Whatman No. 1 filter paper, and the filtrate was collected in a 50-mL volumetric flask. The filtrate was concentrated to dryness using a rotary evaporator at 50°C and 180 rpm. The obtained dry extract was reconstituted in 10 mL of methanol and homogenized. Finally, the solution was passed through a 0.45-μm membrane filter and prepared for HPLC analysis.

#### 2.1.2 HPLC analysis of phenolic compounds

**FIGURE 1:**
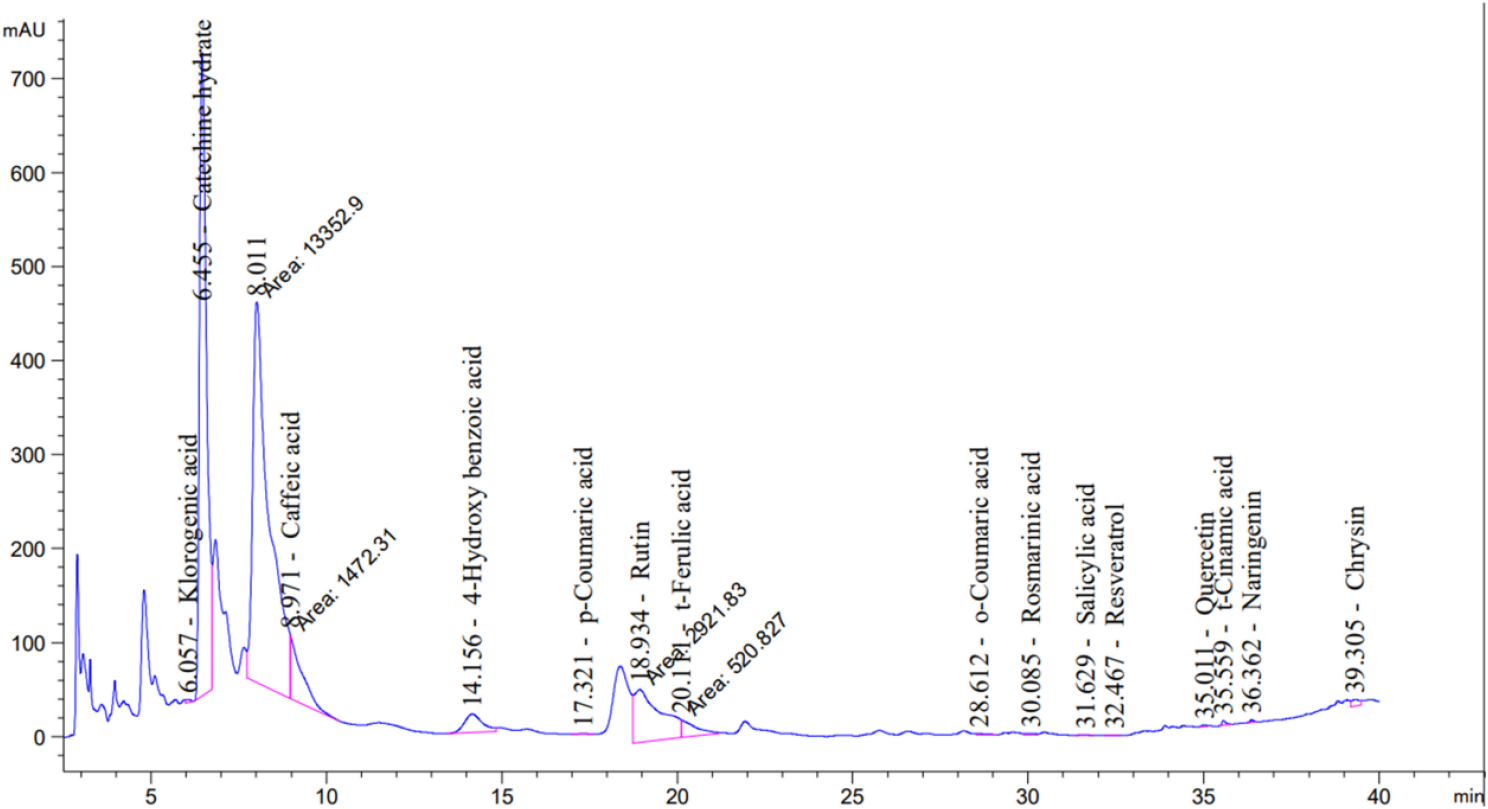
Typical HPLC-DAD chromatogram of phenolic compounds in Matcha tea (280 nm).

Quantitative determination of phenolic compounds was performed using an Agilent 1,260 Infinity HPLC system equipped with a diode-array detector (DAD). For extraction, 250 mg of matcha powder was accurately weighed and extracted with 25 mL of 2% phosphoric acid/ethanol (1:1, v/v) mixture at 30°C for 60 min. The extract was filtered, diluted with water, and analyzed by HPLC. Separation was achieved on an ACE Generix C18 column (250 × 4.6 mm, 5 μm; Advanced Chromatography Technologies Ltd., Aberdeen, Scotland). The column temperature was maintained at 30°C, and the flow rate was set to 0.80 mL/min. The mobile phase consisted of ultrapure water containing 0.1% phosphoric acid (Phase A) and 100% acetonitrile (Phase B). The separation was conducted using the following gradient program: 0 min, 17% B; 7 min, 15% B; 20 min, 20% B; 25 min, 24% B; 28 min, 30% B; 30 min, 40% B; 32 min, 50% B; 36 min, 70% B; and 40 min, returning to 17% B. A 10-μL sample was injected, and detection was performed at 280, 320, and 360 nm. Calibration curves were constructed for each compound in the concentration range of 2.5–250 mg/L, and the linear regression coefficients (*R*^2^) were > 0.99. The HPLC-DAD chromatogram of the matcha tea samples obtained under the described conditions is shown in Figure 1. The major peaks were identified as catechin, rutin, caffeic acid, and chrysin, respectively, while other phenolic acids and flavonoids were also identified according to their characteristic retention times. The results are expressed as mg/g (Supplementary Table 1) (19).

### 2.2 Ethical approval

All experimental procedures were approved by the Tekirdag Namık Kemal University Animal Experiments Local Ethics Committee (approval number: T2024-2192; date: 17.10.2024) and were performed in accordance with the National Institutes of Health guidelines for the care and use of laboratory animals and the ARRIVE reporting standards.

### 2.3 Animals and housing

Thirty-two female Sprague–Dawley rats (10–12 weeks old; 150–210 g) were obtained from the Research and Application Center for Laboratory Animals, Tekirdağ Namık Kemal University (Tekirdağ, Türkiye). Animals were acclimatized for 7 days prior to experimentation and housed in polycarbonate cages (four per cage) under controlled environmental conditions (22 ± 2°C; 45–65% relative humidity; 12 h light/dark cycle). Standard laboratory chow and tap water were provided *ad libitum* throughout the study.

### 2.4 Experimental design

Animals were randomly allocated (simple randomization) into four groups (*n* = 8 per group): Control (sham exposure and vehicle), UV (combined UVA + UVB irradiation and vehicle), Matcha (50 mg/kg, administered once daily by oral gavage with sham exposure), UV + Matcha (combined UVA + UVB irradiation with 50 mg/kg matcha administered once daily by oral gavage).

**FIGURE 2:**
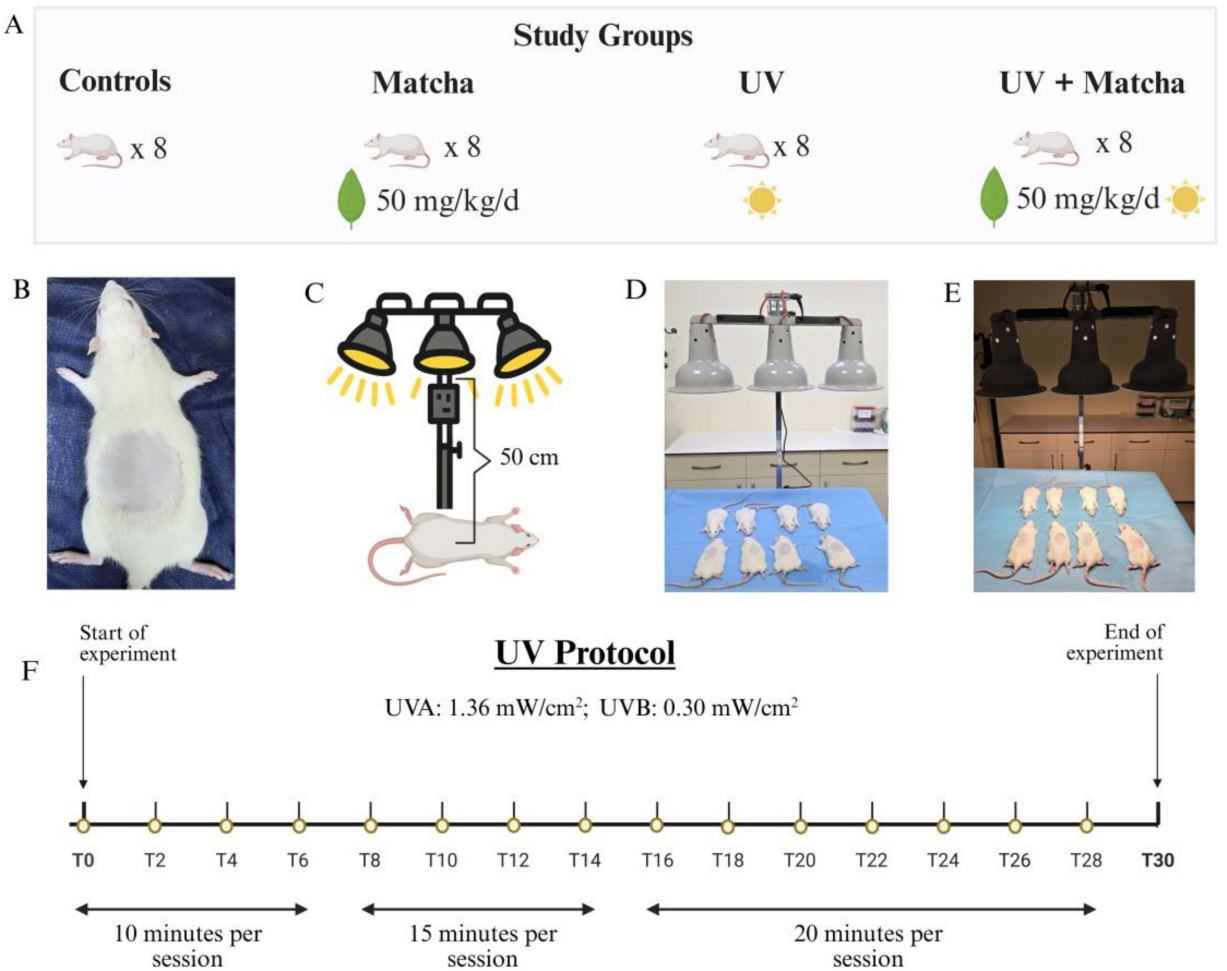
Experimental design and UV irradiation protocol. **(A)** Study groups: Control, UV, Matcha, and UV + Matcha (*n* = 8 per group). Matcha (50 mg/kg) was administered once daily in the Matcha and UV + Matcha groups. Only the UV and UV + Matcha groups received UV irradiation. **(B)** Representative dorsal view of a rat with shaved skin prior to UV exposure. **(C)** Schematic illustration of the UV setup with a fixed 50 cm working distance from the light source to the dorsal skin. **(D)** UV irradiation procedure under standardized conditions. **(E)** UV exposure session in progress. **(F)** Timeline of the UV protocol: 10 min/session during the first week (T0–T6; 4 sessions), 15 min/session during the second week (T8–T14; 4 sessions), and 20 min/session during the third and fourth weeks (T16–T28; 7 sessions). Created in BioRender. Zorlu, Ö. (2026) https://BioRender.com/0mrcv2t.

All groups were handled identically throughout the experimental period. To minimize differential handling- or gavage-related stress, all animals underwent the same daily handling and oral gavage schedule. Vehicle-treated groups received distilled water by oral gavage at volumes equivalent to those used for matcha administration. Thus, any stress associated with handling or gavage was expected to be distributed similarly across groups. Sham exposure replicated the UV procedure with lamps switched off (0 mJ/cm^2^). The experimental workflow is illustrated in Figure 2.

### 2.5 Preparation and administration of matcha

Matcha powder (Toganoo, Istanbul, Turkey; origin: Japan; ceremonial-grade Matcha Gold) was prepared fresh daily by dispersing it in distilled water preheated to 70°C, then allowed to cool to room temperature before administration. Rats in the Matcha and UV + Matcha groups received 50 mg/kg body weight of matcha via oral gavage once daily. The dose was selected to approximate the upper range of habitual human matcha consumption (2–3 g per 60 kg body weight, corresponding to ∼33–50 mg/kg in humans) when expressed on a mg/kg basis (20–22). The administered dose was recalculated individually for each animal prior to each administration based on its current body weight. Control and UV groups received equal volumes of distilled water by gavage to control for handling.

### 2.6 Ultraviolet light exposure

In this study, “UV irradiation” refers to combined UVA + UVB exposure delivered using 300 W Ultra-Vitalux lamps (Osram, Germany) mounted on a floor-based apparatus with three separate lamp sections to ensure uniform exposure (23, 24). The emission spectrum included UVA (315–400 nm) and UVB (285–315 nm) wavelengths.

Prior to irradiation, an approximately 2.5 × 3 cm area of dorsal skin was shaved, with minor variations between animals, to ensure consistent exposure. Animals were anesthetized with ketamine hydrochloride (90 mg/kg, intraperitoneal) and xylazine hydrochloride (10 mg/kg, intraperitoneal) to minimize movement and stress. Lamps were positioned 50 cm above the anesthetized animals.

The UV exposure protocol was adapted from previously established experimental photoaging models, with minor modifications in lamp distance and exposure duration (23–26). UV exposure was administered every other day using a progressive protocol: 10 min per session during week 1, 15 min during week 2, and 20 min during weeks 3–4, for a total of 15 sessions over 30 days. The Control and Matcha groups underwent identical anesthesia and handling procedures under sham exposure (0 mJ/cm^2^) (Figure 2). Exposure duration, lamp distance, animal positioning, anesthesia procedure, and session timing were kept constant across sessions and UV-treated groups.

Direct radiometric measurement with a spectroradiometer was not performed during the experiment. Therefore, UVA and UVB irradiance values were not measured directly at the skin surface but were estimated from the manufacturer-specified technical data for the 300 W Ultra-Vitalux lamp at a 50 cm working distance: 1.36 mW/cm^2^ for UVA and 0.30 mW/cm^2^ for UVB (27). Based on these values and the predefined exposure durations, the estimated per-session doses were 0.816 J/cm^2^ for UVA and 0.180 J/cm^2^ for UVB during week 1 (10 min/session, 4 sessions), 1.224 J/cm^2^ for UVA and 0.270 J/cm^2^ for UVB during week 2 (15 min/session, 4 sessions), and 1.632 J/cm^2^ for UVA and 0.360 J/cm^2^ for UVB during weeks 3–4 (20 min/session, 7 sessions). Accordingly, the cumulative UVA and UVB doses per animal over the 15-session protocol were estimated to be 19.58 J/cm^2^ and 4.32 J/cm^2^, respectively (UVA/UVB = 4.5:1).

Because these calculations were based on manufacturer-specified irradiance values rather than direct skin-surface measurements, the reported UV doses should be interpreted as nominal estimated doses. No experiment-specific numerical uncertainty estimate could be calculated in the absence of direct radiometric measurements. Accordingly, the irradiation protocol represents a standardized repeated-exposure model for controlled between-group comparisons, rather than an individually measured absolute UV dose-response model.

### 2.7 Animal monitoring and welfare assessment

Body weights were measured weekly using a calibrated digital balance ( ± 0.1 g). Animals were observed daily for behavior, grooming, activity level, and any signs of discomfort or visible skin irritation. Humane endpoints were predefined as > 20% body weight loss, persistent hypoactivity, or marked skin damage requiring early euthanasia. UV-induced skin damage was assessed separately by histopathological examination and macroscopic image analysis, as detailed in sections 2.10 and 2.11.

### 2.8 Study termination and sample collection

At the end of the 30-day protocol, all animals were euthanized in compliance with AVMA guidelines by an overdose of pentobarbital sodium, followed by cardiac puncture as a secondary confirmatory method. Blood and skin samples from shaved areas were collected immediately after sacrifice for histological and biochemical analyses. All efforts were made to minimize animal suffering.

### 2.9 Analysis of serum antioxidant markers

Serum levels of catalase (CAT), glucose-6-phosphate dehydrogenase (G6PD), glutathione peroxidase (GPx), glutathione reductase (GR), and superoxide dismutase (SOD) were quantified using commercial enzyme-linked immunosorbent assay (ELISA) kits (Bioassay Technology Laboratory, Shanghai, China; CAT: Cat. No. E0869Ra, G6PD: Cat. No. E0913Ra, GPx: Cat. No. E1242Ra, GR: Cat. No. E1085Ra, SOD: Cat. No. E0168Ra) according to the manufacturer’s instructions.

### 2.10 Histopathological examination

After fixation in 10% neutral buffered formalin for 24 h, skin tissues were washed, dehydrated through a graded ethanol series, and cleared in xylene. The tissues were then embedded in paraffin. Serial 4-μm sections were cut from paraffin blocks, mounted on positively charged slides, and baked at 60°C for 1 h. Sections were deparaffinized in xylene and rehydrated through a descending ethanol series to distilled water. Sections were stained with hematoxylin–eosin (H&E) for general morphological evaluation; elastic van Gieson (EVG; Scytek Laboratories, United States) for visualization of elastic fibers; Masson’s trichrome (Scytek Laboratories, United States) for assessment of collagen fibers; and Picrosirius red (PSR; Scytek Laboratories, United States) for differentiation of collagen types under polarized light. All staining procedures were performed manually according to the manufacturer’s instructions and standard histological protocols. Arterial, muscular, and skin tissues served as positive controls. All slides were evaluated in a blinded manner by a pathologist using an Olympus BX43 microscope (Olympus Corporation, Tokyo, Japan).

### 2.11 Photographic documentation and image analysis

Serial macroscopic photographs of the dorsal skin were obtained weekly during the study for routine visual monitoring of gross changes in skin appearance. For formal macroscopic comparison, standardized endpoint photographs were acquired at study termination to assess erythema, pigmentation, surface roughness, and wrinkle formation using a Samsung Galaxy S25 Ultra (Samsung Electronics, South Korea) mounted on a tripod and positioned perpendicular to the skin surface at a fixed working distance of 20 cm under standardized LED illumination (daylight spectrum, ∼5500 K). Camera settings were kept constant throughout the study with low ISO (∼50–100), *∼1/150 s* shutter speed, fixed focal length, and locked white balance (daylight). All automatic image enhancement and HDR functions were disabled. A color-calibration chart was included in each frame for subsequent normalization of color parameters during analysis.

Histological images were captured using an Olympus BX43 microscope equipped with a DP23 digital camera and cellSens Entry software (version 4.3, Olympus Corporation, Tokyo, Japan). Exposure and white balance were kept constant across sessions. Scale bars shown in histological images were automatically generated in cellSens Entry software based on microscope-camera calibration and represent calibrated real distances (μm), whereas the nominal optical magnification indicates the acquisition setting.

For H&E-stained sections, five separate slides per animal were analyzed. On each slide, five randomly selected non-overlapping fields were photographed at × 200 magnification for morphometric analysis, including epidermal thickness (EpT), papillary dermis thickness (PdT), reticular dermis thickness (RdT), dermal papilla thickness (DpT), epidermal rete ridge thickness (ErT), and interdigitation index (IDI), and at × 400 magnification for epidermal cellularity assessment. One measurement per parameter was obtained from each field, yielding a total of 25 measurements per parameter per animal (5 slides × 5 fields). The mean value per animal was used for statistical analysis. The IDI was calculated as a morphometric ratio that reflects epidermal–dermal interface undulation, as detailed in Supplementary methods.

For Masson’s trichrome-stained sections, images were captured at × 100 magnification, whereas EVG- and PSR-stained slides were imaged at × 200 magnification. For each staining method, five randomly selected non-overlapping fields per slide were photographed across five slides per animal (a total of 25 images per animal). The mean value per animal was used for statistical analysis.

Both macroscopic and histopathological images were analyzed using Fiji (ImageJ, version 2.9.0; National Institutes of Health, Bethesda, MD, United States) (28–30). Pixel-to-micrometer calibration for histopathological images was performed using the *Set Scale* function based on a stage micrometer corresponding to the acquisition magnification. Macroscopic images were calibrated using the embedded reference scale. All measurements were subsequently performed on calibrated images and expressed in micrometers (μm). Skin color was analyzed in the CIE L*a*b* color space (L*: lightness; a*: red–green axis; b*: yellow–blue axis). Detailed step-by-step procedures for H&E morphometry, Masson’s trichrome, EVG, and PSR image analyses, as well as macroscopic texture, wrinkle, and pigmentation analyses, are provided in Supplementary methods.

Quantitative measurements were independently performed by two blinded assessors (a dermatologist with 10 years of experience and a pathologist with 15 years of experience). For all analyses, final values represent the mean of the two raters’ initial measurements. Intra- and inter-rater reliability were evaluated using the intraclass correlation coefficient (ICC). For intra-rater reliability, each rater repeated the measurements after 1 week; repeat measurements were used exclusively for reliability assessment and were not included in the final analytical values.

### 2.12 Statistical analysis

SPSS version 25 (IBM Corp., Armonk, NY, United States) was used for all statistical analyses. Graphs were generated using GraphPad Prism version 11.0.0 (GraphPad Software, San Diego, CA, United States). Continuous variables are presented as median (interquartile range) or mean ± standard deviation, depending on data distribution. Normality was assessed using the Shapiro–Wilk test. For comparisons between groups, one-way ANOVA was used for normally distributed data, whereas the Kruskal–Wallis test was used for non-normally distributed data. Effect sizes were calculated for group comparisons, with eta squared (η^2^) reported for one-way ANOVA and epsilon squared (ε^2^) for the Kruskal–Wallis test. Effect sizes were interpreted according to commonly accepted thresholds, with values of approximately 0.01, 0.06, and 0.14 classified as small, medium, and large, respectively (31). When appropriate, *post-hoc* analyses were performed using Tukey’s or Bonferroni’s test following ANOVA, and the Dunn–Bonferroni approach after the Kruskal–Wallis test. Correlations between continuous variables were evaluated using Pearson’s (r) or Spearman’s (rs) correlation coefficients according to data distribution.

Intra- and inter-rater reliability were assessed using the ICC based on a two-way random-effects model with absolute agreement. A *P*-value < 0.05 was considered statistically significant.

## 3 Results

### 3.1 Phenolic composition of matcha tea powder

Matcha tea exhibited a phenolic-rich composition dominated by catechin hydrate (761.23 ± 14.21 mg/g), followed by rutin (177.97 ± 12.70 mg/g). Moderate levels of caffeic acid, chrysin, 4-hydroxybenzoic acid, and *trans*-ferulic acid, as well as lower concentrations of chlorogenic and rosmarinic acids, along with minor phenolics including coumaric acids, salicylic acid, resveratrol, naringenin, and quercetin, were also detected (Supplementary Table 1).

### 3.2 Serum antioxidant profiles of the study groups

**FIGURE 3:**
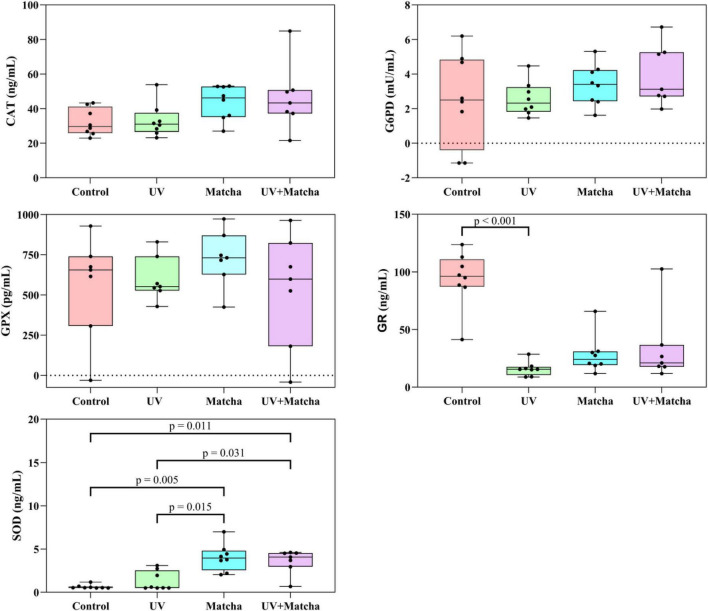
Serum levels of catalase (CAT), glucose-6-phosphate dehydrogenase (G6PD), glutathione peroxidase (GPx), glutathione reductase (GR), and superoxide dismutase (SOD) in the experimental groups.

Serum antioxidant enzyme levels of the study groups are presented graphically in Figure 3, and the corresponding numerical values and statistical details are provided in Supplementary Table 2. Overall group comparisons showed no significant differences in CAT, G6PD, or GPx levels (*p* = 0.067, 0.369, and 0.522, respectively). In contrast, GR levels differed significantly among groups (*p* < 0.001, ε^2^ = 0.589), with lower values in the UV group than in the Control group (*p* < 0.001). No significant difference was observed between the UV and UV + Matcha groups.

SOD levels also varied significantly among groups (*p* < 0.001, ε^2^ = 0.597). Both the Matcha and UV + Matcha groups showed higher SOD levels than the Control group (*p* = 0.005 and *p* = 0.011, respectively) and the UV group (*p* = 0.015 and *p* = 0.031, respectively).

### 3.3 Body weight monitoring

Body weight increased progressively in all groups throughout the study (Supplementary Figure 1). Significant between-group differences in *post-hoc* analysis were limited mainly to the Matcha and UV + Matcha groups. No weight loss or overt signs of systemic toxicity were observed in any group.

### 3.4 Histological and morphometric findings in skin tissue

**FIGURE 4:**
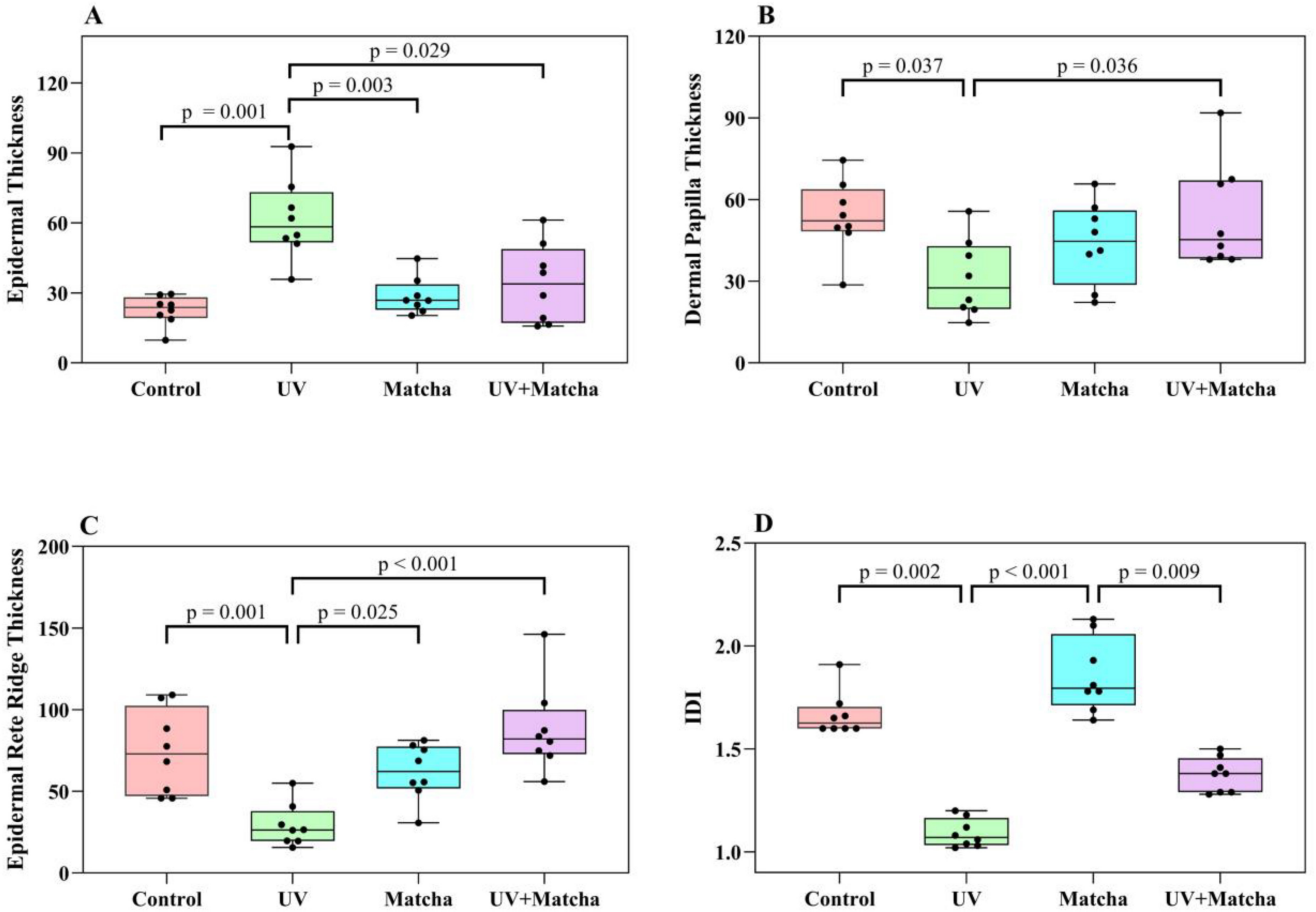
Quantitative analysis of **(A)** epidermal thickness, **(B)** dermal papilla thickness, **(C)** epidermal rete ridge thickness, and **(D)** interdigitation index (IDI) in the Control, UV, Matcha, and UV + Matcha groups.

**FIGURE 5:**
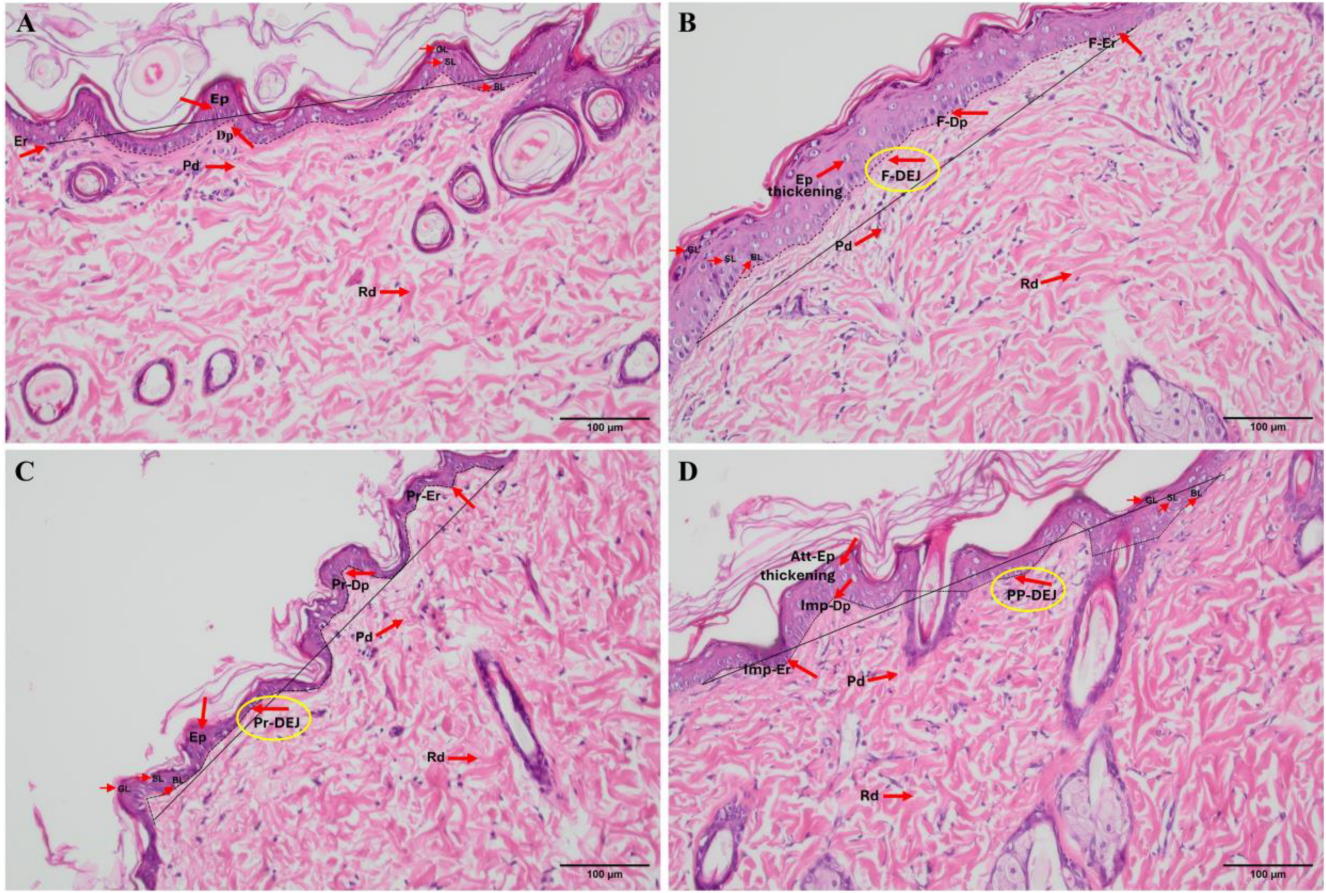
Representative hematoxylin and eosin (H&E)–stained sections of dorsal skin from the Control **(A)**, UV **(B)**, Matcha **(C)**, and UV + Matcha **(D)** groups. The epidermis (Ep), papillary dermis (Pd), reticular dermis (Rd), dermal papilla (Dp), and epidermal rete ridge (Er), and epidermal sublayers, including the basal layer (BL), spinous layer (SL), and granular layer (GL), are indicated by arrows. The dashed line traces the dermoepidermal junction, and the solid line represents the straight-line reference used for interdigitation index (IDI) calculation as the ratio of interface length to straight-line distance. Histological alterations, including epidermal thickening, flattening of the epidermal rete ridges, reduced prominence of dermal papillae, and loss of dermoepidermal junction undulation (reflected by reduced IDI), were more evident in the UV group and appeared attenuated or partially preserved in the UV + Matcha group. Original magnification, × 200; scale bar = 100 μm. F-Er, flattening of epidermal rete ridge; F-Dp, flattening/reduced prominence of dermal papilla; F-DEJ, flattening/loss of dermoepidermal junction undulation; Pr-Er, preserved epidermal rete ridge; Pr-Dp, preserved dermal papilla; Pr-DEJ, preserved dermoepidermal junction undulation; Att-Ep thickening, attenuated epidermal thickening; Imp-Er, improved epidermal rete ridge; Imp-Dp, improved dermal papilla; PP-DEJ, partial preservation of dermoepidermal junction undulation.

Quantitative histomorphometric analyses revealed significant group-dependent alterations across multiple epidermal and dermal parameters (Figure 4 and Supplementary Table 3). Representative H&E-stained sections are shown in Figure 5. EpT differed significantly among groups (*p* < 0.001, η^2^ = 0.596), with higher EpT in the UV group than in the Control group, whereas the UV + Matcha group showed lower EpT than the UV group (*p* = 0.029). DpT and ErT also exhibited significant group effects (*p* = 0.023, η^2^ = 0.284 and *p* < 0.001, η^2^ = 0.537, respectively), with higher values in the UV + Matcha group than in the UV group (*p* = 0.036 and *p* < 0.001, respectively). The IDI demonstrated the strongest group effect (*p* < 0.001, ε^2^ = 0.884), with the UV group showing markedly lower values than the Control and Matcha groups, whereas the UV + Matcha group showed intermediate values.

Granular cell density differed significantly among groups (*p* = 0.023, η^2^ = 0.213), with higher values in the UV + Matcha group than in the Control group (*p* = 0.032). Spinous and basal cell densities did not differ significantly among groups (both *p* = 0.133).

### 3.5 Extracellular matrix evaluation

Overall, extracellular matrix-related parameters showed large group effects, particularly for Masson’s trichrome-stained area (η^2^ = 0.586), PSR total collagen (η^2^ = 0.424), type I collagen (η^2^ = 0.671), type III collagen (ε^2^ = 0.506), and the type I/III collagen ratio (ε^2^ = 0.657).

**FIGURE 6:**
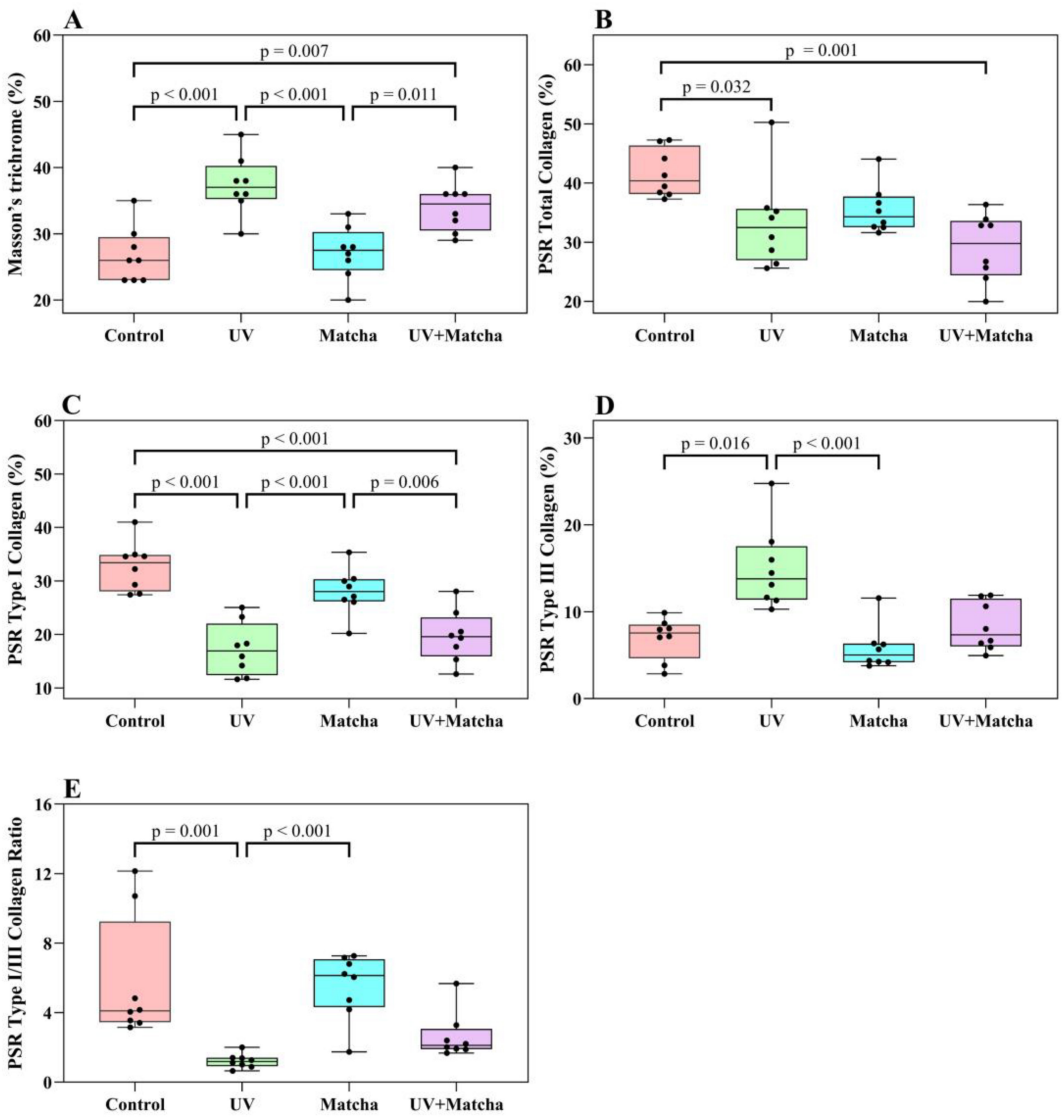
Quantitative analysis of dermal collagen deposition in the Control, UV, Matcha, and UV + Matcha groups. **(A)** Masson’s trichrome–stained area (%), **(B)** PSR total collagen (%), **(C)** PSR type I collagen (%), **(D)** PSR type III collagen (%), and **(E)** PSR type I/III collagen ratio. PSR, Picrosirius Red.

**FIGURE 7:**
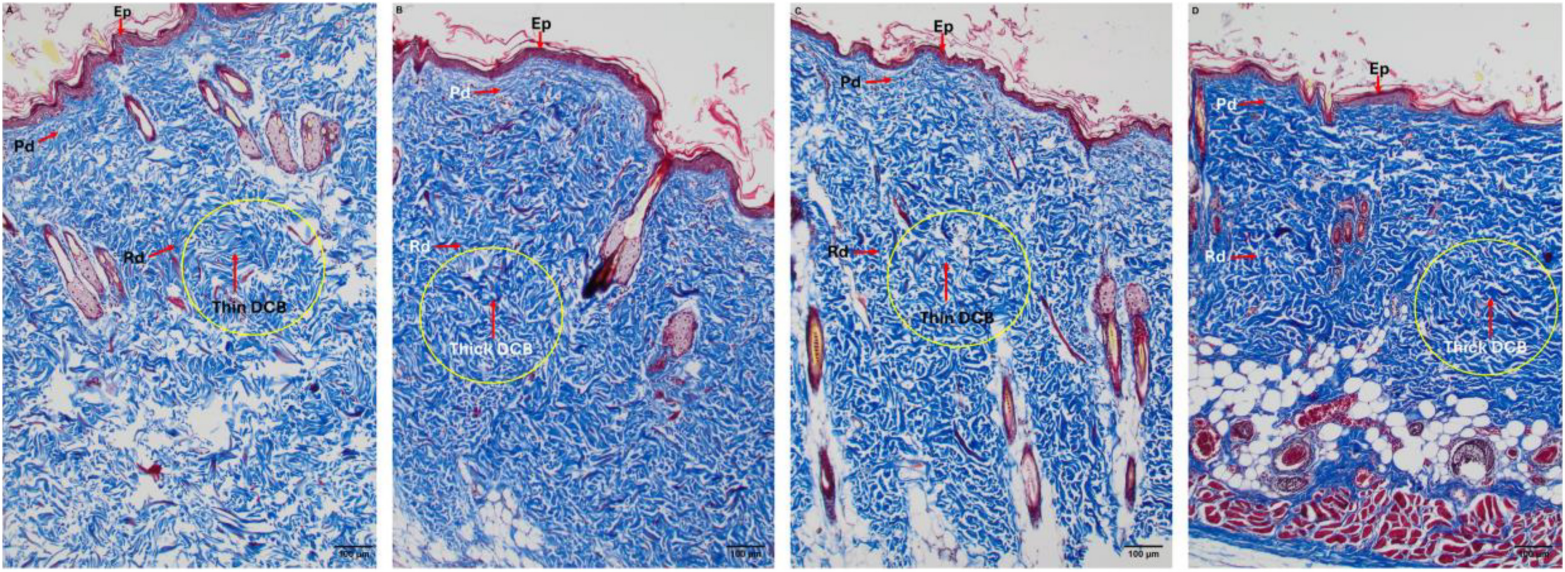
Representative Masson’s trichrome–stained sections of dorsal skin from the Control **(A)**, UV **(B)**, Matcha **(C)**, and UV + Matcha **(D)** groups. Collagen fibers are stained blue. The epidermis (Ep), papillary dermis (Pd), and reticular dermis (Rd) are indicated by arrows. Representative qualitative differences in dermal collagen bundle morphology are shown within the circled areas, with relatively thinner dermal collagen bundles in the Control and Matcha groups and thicker dermal collagen bundles in the UV and UV + Matcha groups. Original magnification, × 100; scale bar = 100 μm. Thin DCB, thin dermal collagen bundles; Thick DCB, thick dermal collagen bundles.

Masson’s trichrome staining demonstrated a significantly higher stained area in the UV group than in the Control and Matcha groups (*p* < 0.001 for each). The UV + Matcha group showed intermediate values, differing significantly from both the Control (*p* = 0.007) and Matcha groups (*p* = 0.011) (Figure 6 and Supplementary Table 4). Representative Masson’s trichrome-stained sections illustrating these changes are shown in Figure 7.

**FIGURE 8:**
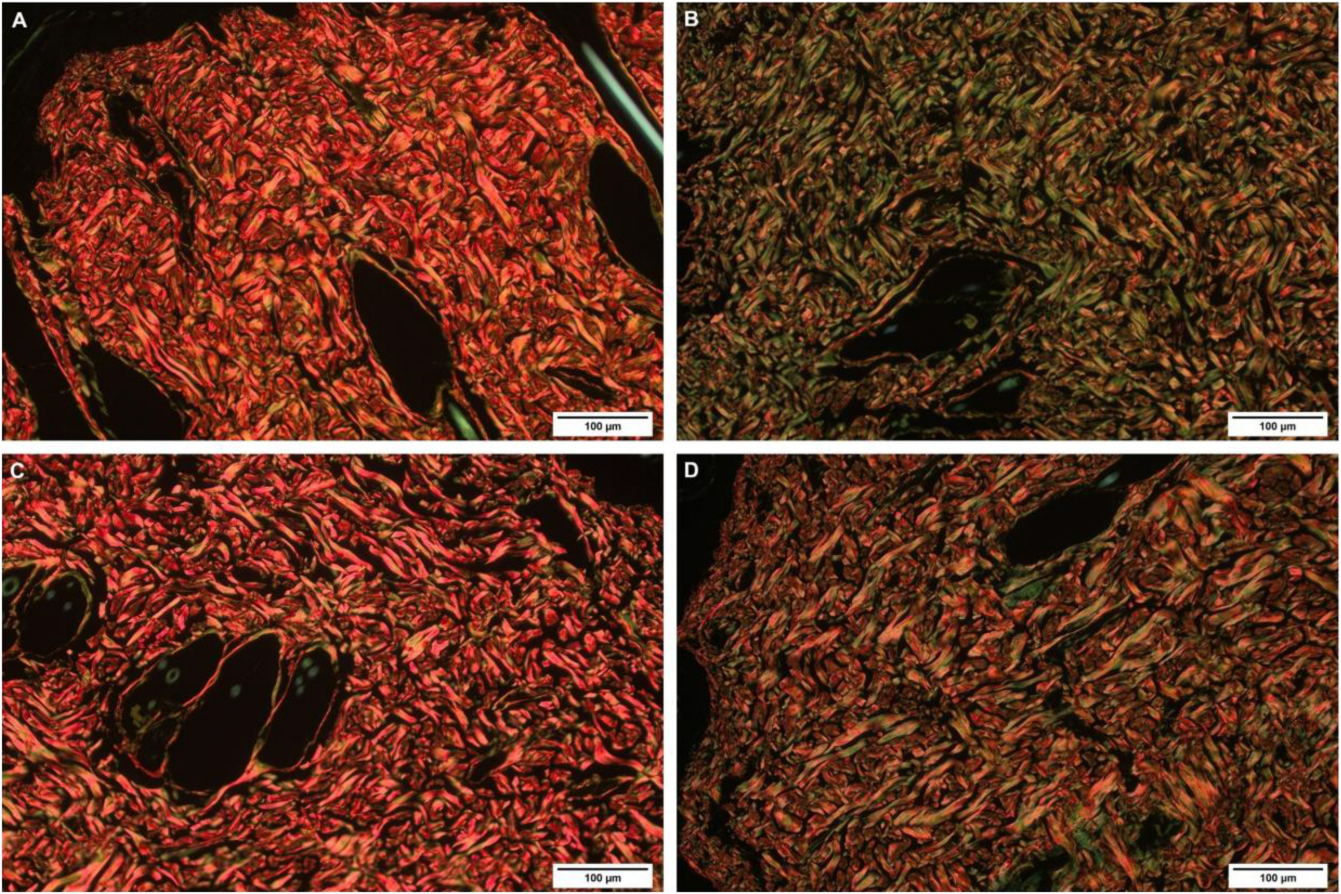
Representative picrosirius red–stained sections of dorsal skin from the Control **(A)**, UV **(B)**, Matcha **(C)**, and UV + Matcha **(D)** groups, examined under polarized light microscopy. Type I collagen fibers appear as red–orange birefringent structures, whereas type III collagen fibers appear greenish. Representative group-dependent differences in collagen birefringence are visible, with relatively greater red-orange signal in the Control and Matcha groups, a more prominent greenish signal in the UV group, and an intermediate birefringence pattern in the UV + Matcha group. Original magnification, × 200; scale bar = 100 μm.

In PSR analysis, total collagen percentage was lower in the UV and UV + Matcha groups than in the Control group (*p* = 0.032 and *p* = 0.001, respectively). Analysis of collagen subtypes showed a marked reduction in type I collagen and a significant increase in type III collagen in the UV group compared with the Control group (*p* < 0.001 and *p* = 0.016, respectively). Consequently, the type I/III collagen ratio was significantly lower in the UV group (*p* = 0.001). The UV + Matcha group showed numerically higher type I collagen values and a higher type I/III collagen ratio than the UV group; however, these pairwise differences did not reach statistical significance. Overall, PSR-based collagen phenotype analysis indicated significant group-dependent alterations, whereas the Control and Matcha groups showed generally similar collagen profiles. Quantitative data are presented in Figure 6 and Supplementary Table 4, and representative PSR-stained sections are shown in Figure 8.

**FIGURE 9:**
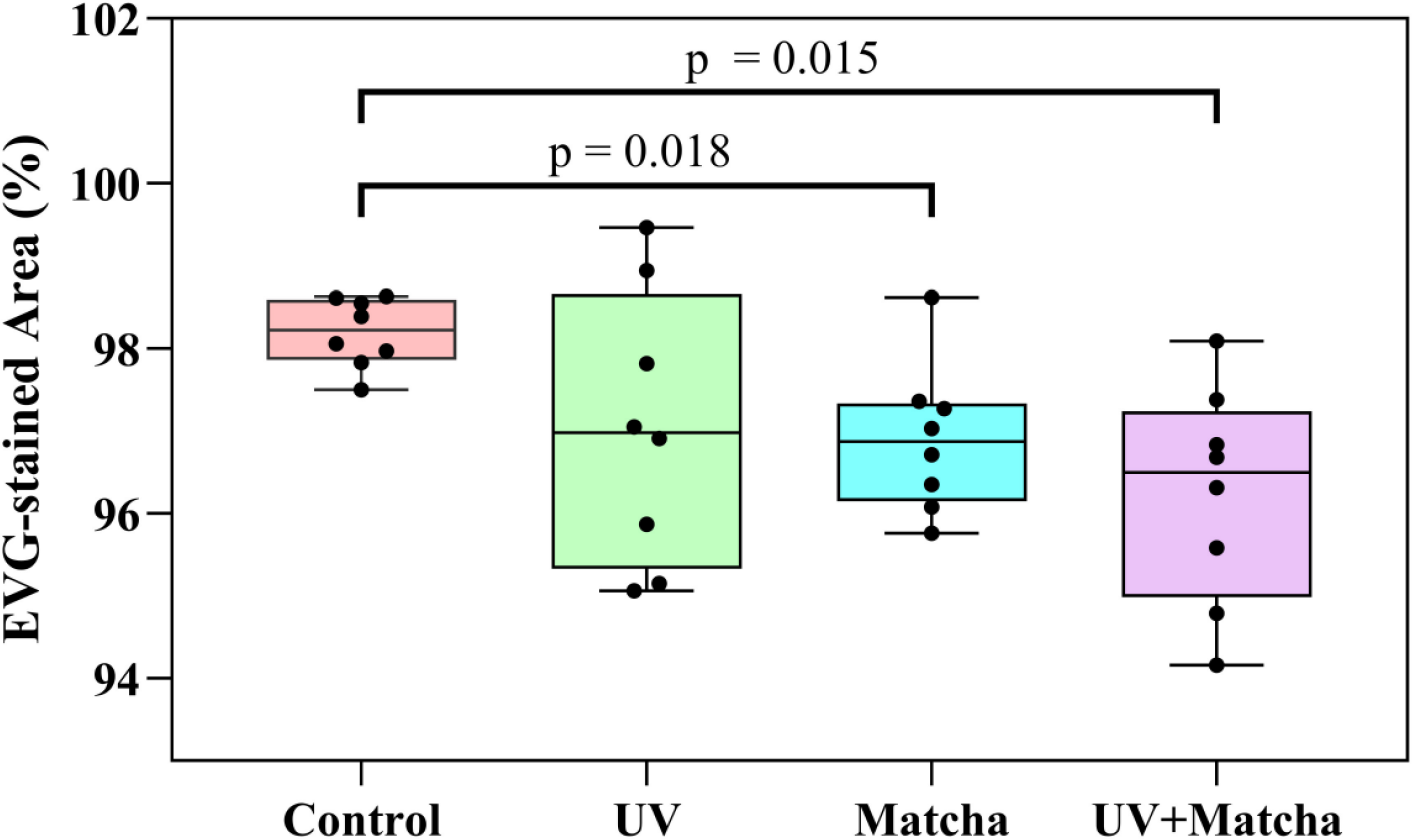
Quantitative analysis of EVG-stained area (%) in the Control, UV, Matcha, and UV + Matcha groups. EVG, Elastic van Gieson staining.

**FIGURE 10:**
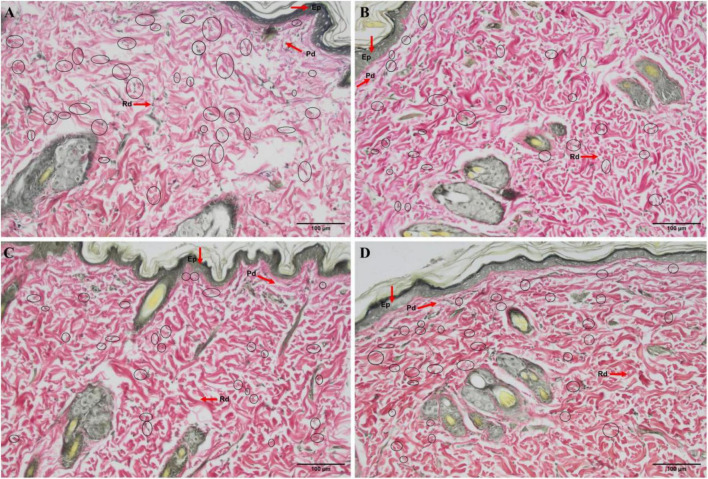
Representative elastic van Gieson (EVG)–stained sections of dorsal skin from the Control **(A)**, UV **(B)**, Matcha **(C)**, and UV + Matcha **(D)** groups. The epidermis (Ep), papillary dermis (Pd), and reticular dermis (Rd) are indicated by arrows. Representative elastic fibers are marked with circles. Elastic fibers appear as dark black structures within the dermis, enabling assessment of elastin organization and distribution. Original magnification, × 200; scale bar = 100 μm.

The EVG-stained area (%) differed significantly among groups (*p* = 0.002, η^2^ = 297), with lower values in the Matcha and UV + Matcha groups than in the Control group (*p* = 0.018 and *p* = 0.015, respectively) (Figure 9 and Supplementary Table 4). Representative EVG-stained sections illustrating elastin fiber organization are shown in Figure 10.

### 3.6 Macroscopic changes in skin appearance

**FIGURE 11:**
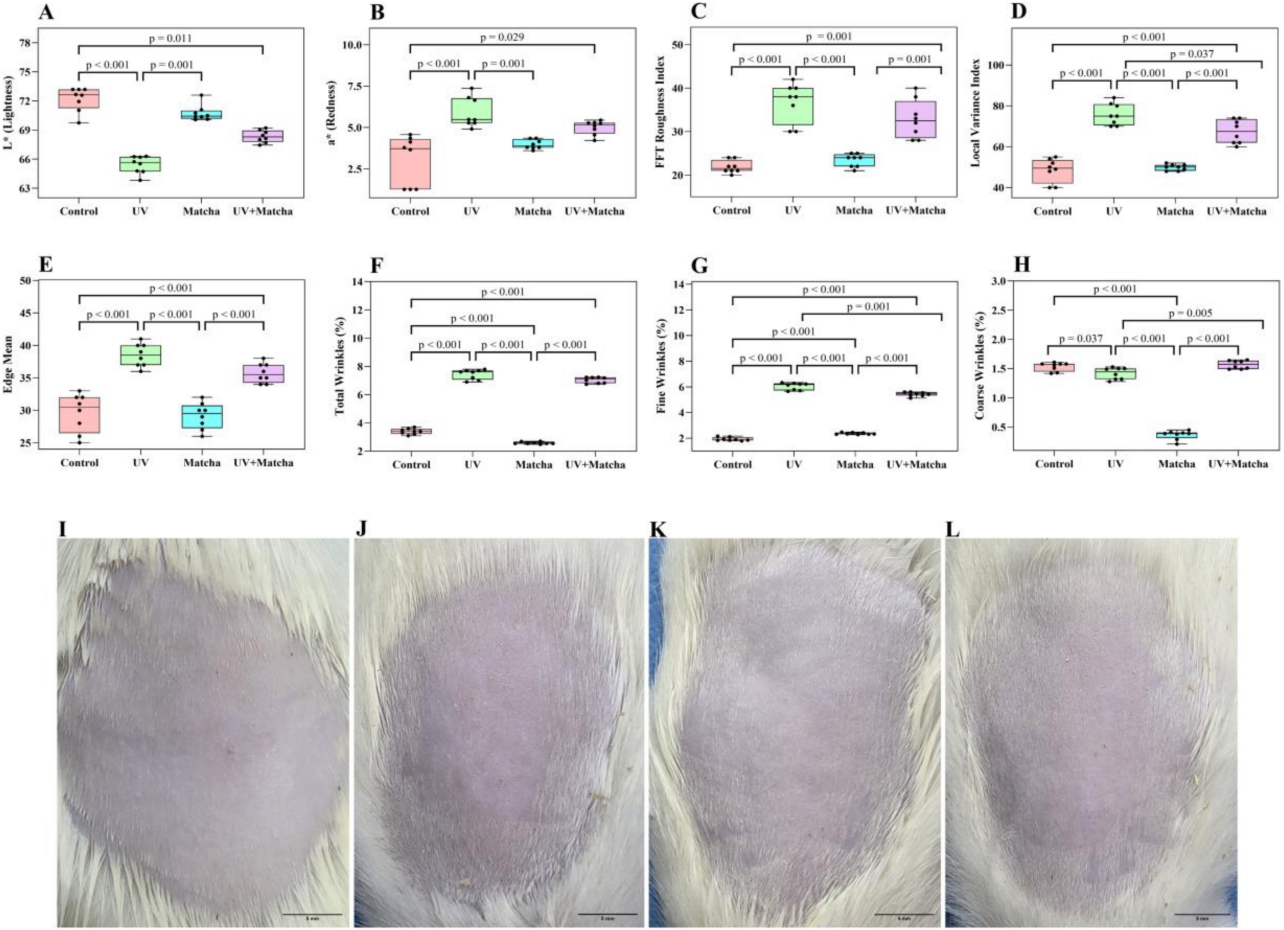
Quantitative macroscopic skin analysis in the Control, UV, Matcha, and UV + Matcha groups. **(A)** L* (lightness), **(B)** a* (redness), **(C)** FFT roughness index, **(D)** local variance index, **(E)** edge mean, **(F)** total wrinkles (%), **(G)** fine wrinkles (%), and **(H)** coarse wrinkles (%). **(I–L)** Representative endpoint dorsal skin photographs from the Control **(I)**, UV **(J)**, Matcha **(K)**, and UV + Matcha **(L)** groups. Macroscopic comparisons were based on standardized quantitative image-analysis metrics derived from the endpoint photographs. FFT, Fast Fourier Transform.

Macroscopic skin parameters are summarized in Figure 11 and Supplementary Table 5. Skin lightness (L*) differed significantly among groups (*p* < 0.001, ε^2^ = 0.872), with lower values in the UV group compared with the Control and Matcha groups (*p* < 0.001 and *p* = 0.001, respectively), consistent with increased skin darkening. The Matcha group showed L* values comparable to the Control group. Although the UV + Matcha group showed higher L* values than the UV group, this difference did not reach statistical significance.

The a* parameter also differed significantly among groups (*p* < 0.001, ε^2^ = 0.734), with higher values in the UV and UV + Matcha groups than in the Control group (*p* < 0.001 and *p* = 0.029, respectively), reflecting increased skin redness. The Matcha group showed values comparable to the Control group. No significant group differences were observed for the b* parameter (*p* = 0.566).

Surface texture analysis also showed significant group-dependent alterations. Both the FFT roughness index and local variance index were higher in the UV group than in the Control and Matcha groups (all *p* < 0.001), indicating increased surface irregularity. The Matcha group showed values similar to the Control group, whereas the UV + Matcha group showed a lower local variance index than the UV group (*p* = 0.037), although values remained above those of the Control group (Supplementary Table 5). Higher edge mean values were also observed in the UV and UV + Matcha groups compared with the Control and Matcha groups (all *p* < 0.001). Effect sizes were large for FFT roughness index, local variance index, and edge mean (η^2^ = 0.803, η^2^ = 0.865, and η^2^ = 0.800, respectively).

Wrinkle-related parameters differed significantly among groups, including total, fine, and coarse wrinkle percentages (all *p* < 0.001; η^2^ = 0.990, η^2^ = 0.991, and η^2^ = 0.977, respectively). The UV group had the highest total and fine wrinkle percentages, whereas the Matcha group had the lowest total wrinkle percentage. The UV + Matcha group showed lower fine-wrinkle percentages than the UV group (*p* = 0.001) but remained higher than the Control group. Coarse wrinkle percentage was lowest in the Matcha group (*p* < 0.001) (Supplementary Table 5).

Representative endpoint dorsal skin photographs are shown in Figures 11I–L; however, macroscopic group differences were assessed primarily by standardized quantitative image-analysis metrics rather than by visual inspection alone. Intra- and inter-rater ICC analyses demonstrated good-to-excellent agreement across all histomorphometric and macroscopic parameters, supporting the reliability of the quantitative assessments (Supplementary Tables 3–5).

### 3.7 Correlation between histological and macroscopic findings

Correlation analyses identified significant associations between antioxidant enzyme levels and selected histological and macroscopic parameters (Supplementary Tables 6–8). SOD levels correlated positively with spinous cell density (r_*s*_ = 0.555, *p* = 0.001), indicating a potential association between serum SOD levels and epidermal cellular integrity. In addition, SOD showed a significant negative correlation with EVG-stained area (%) (r_*s*_ = −0.419, *p* = 0.019). GR also showed correlations with multiple histological and macroscopic parameters (Supplementary Table 6), although group-wise differences were limited.

Furthermore, surface roughness indices, including FFT roughness, local variance, and edge mean, were negatively correlated with IDI, PSR-based type I collagen, and the type I/III collagen ratio, whereas type III collagen was positively correlated with these roughness parameters, reflecting a shift toward immature reparative collagen in photodamaged skin. Total and fine wrinkle percentages were also negatively correlated with IDI (r_*s*_ = −0.838 and r_*s*_ = −0.744, respectively; both *p* < 0.001) and PSR-based type I/III collagen ratio (r_*s*_ = −0.700 and r_*s*_ = −0.704, respectively; both *p* < 0.001).

Overall, these findings suggest that microscopic alterations in dermoepidermal architecture and collagen phenotype are associated with quantitative macroscopic measures of skin surface deterioration.

## 4 Discussion

### 4.1 Principal findings

In this study, we investigated the effects of oral supplementation with whole-leaf matcha powder on skin photoaging using a well-established rat model that integrates biochemical, histological, and macroscopic endpoints. The principal findings indicate that matcha supplementation was associated with partial phenotypic attenuation of selected UV-induced changes, particularly at the level of dermoepidermal architecture, collagen phenotype, and skin surface texture. Specifically, matcha supplementation was associated with higher serum SOD levels, attenuation of UV-induced epidermal thickening, partial improvement in ErT and dermal papilla structure, a numerical shift toward a higher type I/III collagen ratio, and improvement in selected surface parameters, including surface roughness indices and fine wrinkle percentage. Collectively, these findings indicate that oral matcha supplementation was associated with phenotypically relevant but incomplete attenuation of selected UV-induced changes, supporting its potential role as a dietary adjunct for mitigating photoaging-related alterations. Importantly, these findings should be interpreted as phenotypic and exploratory, because molecular markers and signaling pathways related to oxidative stress, inflammation, and extracellular matrix turnover were not directly assessed.

### 4.2 UV-induced photoaging is characterized by structural disruption and collagen remodeling

UV-induced photoaging reflects the convergence of DNA damage, oxidative stress, inflammatory signaling, and extracellular matrix degradation. UV radiation increases ROS generation, leading to lipid and protein oxidation and activation of redox-sensitive pathways, including AP-1, MAPK, and NF-κB. These events promote MMP upregulation, inflammatory cytokine production, and impaired TGF-β-dependent collagen homeostasis, resulting in progressive collagen degradation, abnormal elastic fiber remodeling, and extracellular matrix disorganization. In parallel, accumulation of unrepaired DNA damage may contribute to genomic instability, cell cycle arrest, and apoptosis. Collectively, these processes manifest as epidermal thickening, collagen remodeling, elastotic change, and visible surface deterioration (1–3, 32–34).

Consistent with established models of photoaging, repeated UV exposure in the present study induced pronounced alterations across multiple structural levels of the skin (1, 3, 33, 34). These changes included epidermal hyperplasia, flattening of rete ridges, dermal papilla atrophy, a marked reduction in the IDI, and substantial remodeling of the dermal extracellular matrix.

Photoaged skin is histologically characterized by modifications in extracellular matrix composition, abnormal elastin accumulation, increased collagen degradation, and impaired collagen synthesis (1, 2, 33–35). Previous studies have shown that UV irradiation disrupts collagen homeostasis by suppressing mature type I collagen and shifting the dermal matrix toward a relatively less mature collagen phenotype, often accompanied by a reduced type I/III collagen ratio (36–38). In addition, type III collagen has been reported to exert protective effects in UV-induced skin photoaging models, including attenuation of epidermal and dermal thickening, enhancement of type I and type III collagen secretion, extracellular matrix remodeling, and suppression of UVB-induced autophagic responses in human keratinocytes (39, 40).

Consistent with these observations, the present findings showed that UV exposure was associated with a shift in collagen composition toward increased type III collagen and a substantially reduced type I/III ratio. These histological alterations were paralleled by macroscopic manifestations of photoaging, including increased surface roughness metrics, elevated wrinkle percentages, and altered skin color parameters, underscoring the close relationship between microstructural damage and visible skin aging phenotypes. The large effect sizes observed for IDI, EpT, collagen phenotype, and wrinkle-related parameters further emphasize the robustness of UV-induced phenotypic damage in this model and provide a stringent framework for evaluating interventions aimed at attenuating UV-induced photoaging-related changes. Although these findings are biologically consistent with known UV-related pathways, molecular markers of extracellular matrix turnover, oxidative stress, and inflammation were not directly assessed; therefore, the observed changes should be interpreted as phenotypic rather than mechanistic evidence.

### 4.3 Matcha supplementation was associated with selective changes in serum antioxidant enzyme profiles

Matcha tea is widely recognized for its high antioxidant content, largely attributable to its rich polyphenolic and catechin composition (4, 41). Although human clinical evidence remains limited and the oxidative processes examined vary among studies, accumulating animal and *in vitro* data support the notion that matcha supplementation can ameliorate oxidative stress and inflammatory responses in diverse experimental models. A recent meta-analysis of randomized controlled trials in humans reported that green tea supplementation increased SOD and GPx levels, although it had no significant effect on most inflammatory markers, except IL-1β (42). Experimental studies have likewise shown that catechin-based interventions can mitigate oxidative stress and enhance endogenous antioxidant defenses. For example, Al-Zharani et al. reported that catechins attenuated cadmium-induced oxidative stress in rats and increased levels of glutathione, SOD, GPx, and catalase (43), whereas Oyovwi et al. showed that epigallocatechin gallate ameliorated polystyrene microplastics-induced oxidative stress and increased serum SOD, CAT, and GR activities in rats (44). Matcha-specific studies also support this antioxidant potential. Xu et al. reported that matcha administration improved oxidative status, including SOD, GPx, and malondialdehyde-related parameters, in mice fed a high-fat diet (45). Similarly, matcha was shown to enhance antioxidant enzymes, including SOD, GPx, and glutathione transferase, in rats fed a high-fat-sucrose diet (46). In contrast, clinical studies have reported more variable findings. Green tea consumption showed no significant effect on serum GPx and CAT activities in adults with metabolic syndrome (47). Similarly, matcha supplementation did not significantly alter SOD, GPx, and CAT levels in overweight or obese individuals (48).

In contrast to the broader antioxidant responses reported in other models, the serum antioxidant enzyme profile observed in the present study appeared more selective. Among the enzymes examined, SOD showed the most consistent difference associated with matcha administration, whereas CAT, G6PD, and GPx did not differ significantly among groups. Notably, GR activity was markedly lower in the UV group than in the Control group and was not significantly restored in the UV + Matcha group. This divergence between higher SOD activity and limited GR recovery indicates an enzyme-specific serum antioxidant response rather than broad normalization of the measured antioxidant profile under the present experimental conditions. The increase in SOD may reflect a compensatory response to altered redox status, but it should not be interpreted as evidence of systemic antioxidant protection in the absence of parallel recovery in GR and other downstream antioxidant parameters. However, because tissue-level oxidative damage markers, redox-sensitive transcription factors, and molecular antioxidant pathways were not assessed, these findings should be interpreted as exploratory biochemical observations rather than direct evidence of molecular regulation of oxidative stress pathways. Further molecular-level studies are required to determine whether these serum antioxidant enzyme changes are linked to the observed partial attenuation of UV-induced skin changes.

### 4.4 Matcha supplementation was associated with partial attenuation of UV-induced epidermal–dermal microarchitectural alterations

One of the most prominent findings of this study is the partial attenuation of UV-induced epidermal–junctional microarchitectural disruption in matcha-supplemented animals. This included significant mitigation of UV-induced reductions in ErT, DpT, and the IDI, changes that are histopathological hallmarks of photoaged skin and are known to compromise mechanical stability, nutrient exchange, and resistance to wrinkle formation (33, 34), with the IDI showing the largest effect size among all histomorphometric parameters.

The functional relevance of these architectural changes is underscored by the strong negative correlations observed between IDI and both surface roughness and wrinkle indices. These associations suggest that greater epidermal–dermal interface complexity may be linked to smoother skin surface characteristics and improved structural resilience.

Importantly, the matcha-associated changes were more evident at the epidermal–junctional microarchitectural level than in deeper dermal parameters, suggesting differential endpoint sensitivity and/or differences in the timing of dermal remodeling processes.

Although UV exposure tended to reduce RdT, matcha supplementation did not attenuate this decrease when administered together with UV. This finding did not parallel the more evident matcha-associated changes observed in epidermal–junctional microarchitectural parameters. Given that RdT may reflect not only collagen content but also inflammation, edema, and extracellular matrix remodeling, the lower RdT observed in the UV + Matcha group may reflect complex and compartment-specific dermal responses rather than a straightforward indication of either structural improvement or enhanced matrix degradation. This discrepancy highlights the need for further investigation using direct assessments of extracellular matrix remodeling, inflammatory status, and edema-related changes in the reticular dermis.

### 4.5 Collagen phenotype changes with matcha supplementation

UV exposure disrupts collagen homeostasis by simultaneously enhancing matrix degradation and impairing TGF-β/Smad-dependent collagen synthesis. UV-induced ROS activate AP-1/MAPK- and NF-κB-related pathways, promoting MMP expression and inflammatory signaling while reducing type I procollagen synthesis. The net result is a loss of mature fibrillar collagen, extracellular matrix disorganization, and a shift toward a less-organized remodeling phenotype characteristic of photoaged skin (32, 49).

The combined application of Masson’s trichrome and PSR staining revealed important qualitative distinctions in collagen remodeling following UV exposure and matcha supplementation. These differences can, in part, be attributed to the distinct analytical properties of the staining methods. Masson’s trichrome lacks specificity for individual collagen subtypes and may overestimate collagen-rich areas in the presence of thin or fragmented fibers, whereas PSR staining, particularly when evaluated under polarized light, is considered a benchmark method for the detection and semi-quantitative assessment of collagen content and for distinguishing type I from type III collagen (30, 50).

UV exposure resulted in an increased Masson’s trichrome-positive area, while PSR analysis demonstrated a reduction in total collagen content. Rather than reflecting a true increase in structurally intact collagen, this discrepancy likely represents UV-induced extracellular matrix remodeling characterized by extensive fragmentation and disorganization of collagen fibers, especially type I. Such degraded fibrils retain affinity for Masson’s trichrome staining, but exhibit reduced birefringence under polarized light, leading to lower PSR-detected collagen levels. Consistent with this interpretation, UV exposure decreased the proportion of type I collagen and the type I/III ratio, while increasing type III collagen, indicating a shift toward a less mature collagen phenotype rather than genuine collagen accumulation.

Matcha supplementation did not restore total collagen content over the experimental period; however, the UV + Matcha group showed a trend toward a more favorable collagen composition, with numerically higher type I collagen, lower type III collagen, and a higher type I/III ratio relative to the UV group. These findings suggest that the matcha-associated changes were more apparent at the level of collagen phenotype than bulk collagen deposition. However, because collagen-related gene or protein expression markers were not assessed, the present data cannot determine whether these differences reflect altered collagen synthesis, degradation, or extracellular matrix remodeling pathways.

Notably, recent experimental studies suggest that the role of type III collagen in photoaging may be context-dependent. Although an increased relative proportion of type III collagen in photodamaged skin may reflect a less mature remodeling phenotype, exogenous type III collagen-based interventions have also been reported to reduce oxidative stress, autophagy, apoptosis, senescence, and matrix disorganization while improving dermal structure in experimental photoaging models (39, 51). These observations suggest that type III collagen may participate in reparative responses during UV-induced matrix remodeling, rather than serving solely as a passive indicator of immature collagen deposition.

### 4.6 Elastic fiber–related changes reflect matrix organization rather than elastin loss

Because UV exposure promotes extracellular matrix remodeling and elastotic degeneration, elastic fiber–related histological signals may reflect changes in fiber organization and matrix context rather than absolute elastin abundance alone (2, 32). Accordingly, the reduced EVG signal observed in matcha-supplemented groups should be interpreted with caution. Rather than necessarily indicating true elastin depletion, the observed reduction may reflect matrix compaction, alterations in fiber spatial organization, or optical variations secondary to changes in collagen architecture and overall dermal composition. Given the semi-quantitative nature of EVG-based image analysis, staining variability, stromal context, section quality, and image-processing parameters, including segmentation strategy and threshold selection, may influence signal detection independent of absolute elastin content (52, 53).

Shape-related parameters (circularity and solidity) did not differ significantly among groups. However, these metrics were calculated from aggregated elastin-positive regions derived from binary masks rather than from individually delineated elastic fibers. Therefore, the absence of group differences may reflect stability in segmented area morphology and/or segmentation-driven coalescence of adjacent fibers, rather than definitive preservation of single-fiber microarchitecture.

Collectively, these findings suggest that EVG-derived metrics in the present model likely represent changes in matrix organization and spatial distribution of elastin-positive areas rather than isolated alterations in elastin quantity. These considerations underscore the need for cautious interpretation of elastin-related parameters within the context of dynamic extracellular matrix remodeling and semi-quantitative digital image analysis. Future studies incorporating elastin-specific immunohistochemistry, ultrastructural analysis, or biochemical quantification of elastin and its degradation products would help distinguish between structural reorganization, compositional shifts, and true changes in elastin abundance.

### 4.7 Structural changes correlate with macroscopic skin texture and wrinkle formation

Significant correlations were observed between ErT, IDI, the collagen type I/III ratio, and parameters reflecting surface roughness and wrinkle formation. Increased type III collagen content was positively associated with surface irregularity, which may be consistent with a shift toward a less mature collagen phenotype in photodamaged skin (36–38). Alterations in dermoepidermal architecture and loss of mature collagen support would be expected to reduce resistance to surface irregularity and wrinkle formation. Thus, the relative maintenance of IDI and a more favorable collagen phenotype in the UV + Matcha group may help explain the lower roughness and fine-wrinkle values observed in this group.

While these associations do not imply direct causality, they suggest that alterations in skin microarchitecture are closely linked to measurable changes in visible skin texture. The partial improvement in surface roughness and fine-wrinkle parameters in the UV + Matcha group may therefore reflect structural modulation at the tissue level, even in the absence of complete biochemical normalization.

From a translational perspective, these findings suggest that oral matcha supplementation may be associated with subtle improvements in visible skin characteristics, although the underlying oxidative and matrix-related pathways require molecular validation. However, extrapolation to clinical settings should be approached cautiously, and further studies incorporating longitudinal designs and functional biomechanical assessments are needed to clarify the extent to which partial microstructural maintenance translates into durable cosmetic or dermatological benefit.

### 4.8 Implications for dietary photoprotection and comparison with existing green tea literature

Previous studies investigating oral green tea supplementation in the context of photoaging or UV-induced skin injury have primarily focused on concentrated extracts, isolated catechins or flavonoids, short-term UV exposure models, or limited histological endpoints. In several cases, oral supplementation was administered in combination with topical green tea formulations or additional antioxidants, such as vitamin C, thereby limiting the attribution of observed effects specifically to oral green tea intake (9, 10, 18, 54). A meta-analysis of randomized controlled trials reported that oral green tea catechins reduced UV-induced erythema and inflammation and increased the minimal erythema dose in humans (18). More recent systematic reviews have continued to support antioxidant, anti-inflammatory, and DNA-protective mechanisms, while also emphasizing persistent heterogeneity across clinical studies (55). Although polyphenol supplementation showed photoprotective effects in most studies, some investigations failed to demonstrate significant benefit, and dose-dependency was not consistently established (55). In contrast, a double-blind randomized controlled trial found that oral green tea catechins did not confer significant protection against UV-induced erythema or direct DNA damage and could not substitute for topical sunscreen use (56). Sheng et al. reported that topical application of gallocatechin gallate and epigallocatechin gallate attenuated UVB-induced collagen degradation, suggesting a protective role against photoaging by preserving collagen integrity and function (57). However, oral matcha-specific photoprotection data remain limited compared with the larger body of literature on green tea catechins and related polyphenols.

An important translational consideration is that oral and topical photoprotective approaches differ substantially in route of delivery and expected tissue exposure (9, 10, 58, 59). Topical green tea- or catechin-containing formulations may act more directly at the skin surface and superficial cutaneous compartments (58), whereas orally administered matcha depends on gastrointestinal absorption, first-pass metabolism, systemic circulation, and tissue distribution before reaching the skin (9, 10, 59). Therefore, the cutaneous effects of oral supplementation depend not only on the administered dose but also on bioavailability, circulating metabolite profiles, and the extent of skin-level exposure. Although the observed findings are consistent with systemic effects of oral supplementation, the absence of plasma catechin, circulating matcha-derived metabolite, and skin tissue level measurements precludes confirmation of the extent of systemic and cutaneous exposure achieved in this model.

In contrast to prior studies, the present investigation evaluated whole-leaf matcha powder, which provides a broader spectrum of bioactive constituents, under a repeated UV-exposure paradigm designed to model cumulative photoaging-associated alterations in epidermal and dermal microarchitecture, as well as macroscopic changes. Collectively, the findings suggest that oral matcha supplementation may be associated with partial attenuation of selected UV-induced photoaging-related changes, accompanied by partial maintenance of epidermal–junctional microarchitecture and selectively higher serum SOD levels. Plant-based photoprotective approaches may offer potential advantages due to their broader spectrum of bioactive constituents, reported antioxidant and anti-inflammatory properties, and relative affordability. At the same time, their safety, tolerability, and formulation-specific toxicological profile require careful evaluation. Importantly, the present findings are most appropriately interpreted within the framework of oral supplementation and should be considered complementary to, rather than a substitute for, established photoprotective strategies such as topical sunscreen use and behavioral UV avoidance.

### 4.9 Strengths and limitations of the study

The present study possesses several strengths, including the integrated assessment of epidermal–dermal microarchitecture, collagen phenotype, elastic fiber metrics, antioxidant enzyme profiles, and surface-texture parameters within a repeated UV-exposure model, thereby enabling a direct linkage between microscopic alterations and visible photoaging phenotypes. Unlike studies employing isolated catechins or processed green tea extracts, we utilized whole-leaf matcha powder, enabling exposure to the full spectrum of naturally occurring bioactive constituents. This approach may enhance translational relevance by more closely reflecting real-world dietary consumption patterns. In addition, differentiation of collagen subtypes provides phenotypic insight into collagen subtype distribution and qualitative matrix alterations rather than relying solely on bulk collagen estimation. Finally, the observed correlations between structural and macroscopic parameters support the functional relevance of the histological findings. In addition, the high intra- and inter-rater ICC values observed for histomorphometric and image-based parameters support the methodological robustness and reproducibility of the quantitative assessments.

Several limitations should be acknowledged. The study employed a single dose and duration of matcha supplementation and utilized only female rats, which may limit generalizability across sexes and dosing paradigms. Body surface area normalization of the administered dose yields a human equivalent dose of ∼8.1 mg/kg/day (∼0.5–0.6 g/day for a 60–70 kg adult), which should be considered when interpreting translational results (60). However, dose extrapolation based on either body weight or body surface area does not establish pharmacokinetic or bioavailability equivalence between rats and humans. Because plasma catechin concentrations, circulating matcha-derived metabolites, and skin tissue levels were not measured, the extent to which oral matcha supplementation achieved systemic and cutaneous exposure sufficient to affect skin responses cannot be determined from the present study. Daily oral gavage may also represent a procedural stressor that could influence metabolic or systemic oxidative parameters. Although identical handling and vehicle-gavage procedures were applied across groups to minimize differential bias, gavage-related stress cannot be completely excluded as a general confounder, particularly for serum antioxidant measurements. The repeated UV exposure model, while inducing cumulative structural alterations, may not fully recapitulate the long-term complexity of human photoaging. The relative contributions of UVA and UVB were not independently characterized, limiting wavelength-specific mechanistic interpretation. As a major methodological limitation, UVA and UVB dose estimates were derived from manufacturer-specified irradiance values rather than from direct radiometric measurements at the skin surface, which may limit precise quantification of the delivered UV dose under the experimental conditions. Because experiment-specific radiometric data were not obtained, a formal numerical uncertainty estimate for the delivered UVA and UVB doses could not be calculated. Although the irradiation protocol was standardized across UV-treated groups, potential sources of uncertainty include lamp aging, warm-up state, lamp-to-lamp variability, spectral output variation, spatial heterogeneity of the irradiation field, and minor differences in skin surface orientation. Therefore, the reported UVA and UVB doses should be interpreted as nominal estimated doses rather than directly measured skin-surface irradiance values. Molecular-level validation of oxidative stress, inflammatory signaling, and extracellular matrix turnover pathways, including gene- or protein-level assessment, was not performed. Therefore, the present findings should be interpreted as phenotypic and exploratory rather than as direct mechanistic evidence. In addition, assessment of elastic fibers relied on semi-quantitative histomorphometry without complementary biochemical or ultrastructural validation. Finally, systemic levels of matcha-derived constituents were not measured, and gastrointestinal metabolism and epithelial transport processes may limit the extent to which specific compounds reach the skin in biologically active concentrations.

### 4.10 Future directions

Future studies should explore dose–response relationships, longer exposure durations, and potential sex-specific differences to better define the scope and generalizability of matcha’s effects. Gene- and protein-level investigations of MMPs, redox-sensitive transcription factors, inflammatory mediators, and extracellular matrix turnover pathways would further clarify whether the observed structural changes are accompanied by molecular-level alterations in photoaging-related pathways. In addition, assessment of elastin organization using biochemical or ultrastructural approaches and evaluation of biomechanical properties such as skin elasticity and tensile strength may help link histological preservation to functional outcomes. Finally, carefully designed human intervention studies are warranted to determine whether the phenotypic and structural differences observed in this experimental model translates into clinically meaningful effects under real-world photodamage conditions.

## 5 Conclusion

Oral whole-leaf matcha supplementation was associated with partial attenuation of selected UV-induced photoaging-related changes in this repeated UV exposure model. The most notable findings involved partial attenuation of dermoepidermal microarchitecture disruption, a numerical shift toward a higher type I/III collagen ratio, and improvement in selected macroscopic skin parameters, whereas serum antioxidant enzyme changes appeared more limited and selective. These findings suggest a potential adjunctive role for matcha as a dietary photoprotective strategy. However, further molecular-level and translational studies are required to clarify the underlying pathways and establish the clinical relevance of these effects in human photodamage.

## Data Availability

The raw data supporting the conclusions of this article will be made available by the authors, without undue reservation.
