# Supplementary figures and images for: Oral whole-leaf matcha partially attenuates UV-induced dermoepidermal disruption and collagen phenotype alterations in a rat model of repeated photoaging
Source: Front Med (Lausanne). 2026 Jun 10;13:1813454. doi: 10.3389/fmed.2026.1813454 (PMC13290547; doi:10.3389/fmed.2026.1813454)

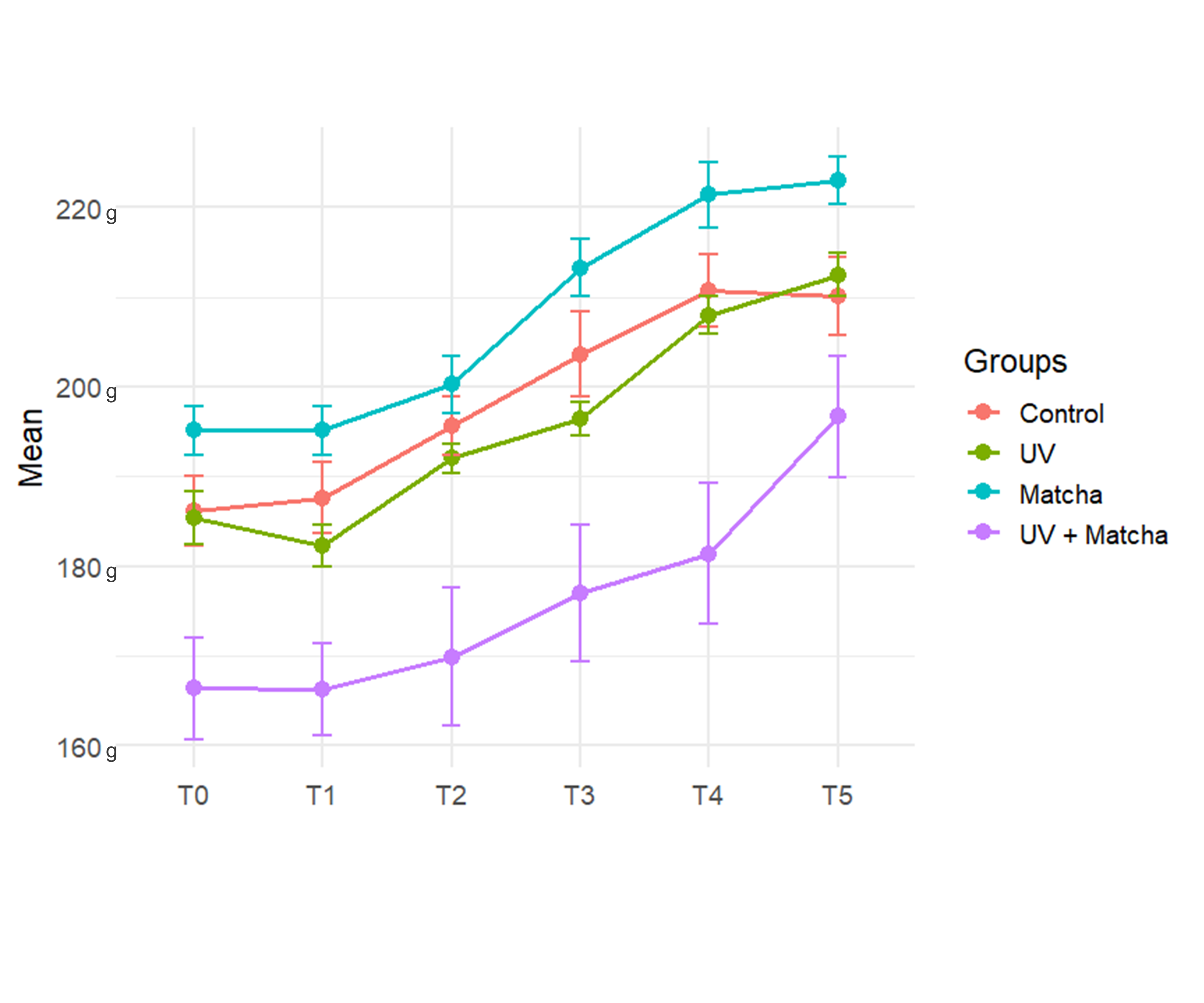

Supplement: Supplementary file 1 [file Image_1.png]
