# Supplementary material for: Oral whole-leaf matcha partially attenuates UV-induced dermoepidermal disruption and collagen phenotype alterations in a rat model of repeated photoaging
Source: Front Med (Lausanne). 2026 Jun 10;13:1813454. doi: 10.3389/fmed.2026.1813454 (PMC13290547; doi:10.3389/fmed.2026.1813454)
Supplement: Supplementary file 2 [file Table_1.docx]

**Supplementary Tables**

**Table S1.** Concentrations of phenolic compounds identified in matcha tea (mg/g).

| **No** | **Phenolic compounds (mg/g)** | **Sample** |
| --- | --- | --- |
| 1 | Catechin hydrate | 761.23 ± 14.21 |
| 2 | Rutin | 177.97 ± 12.7 |
| 3 | Caffeic acid | 40.76 ± 1.27 |
| 4 | Chrysin | 35.14 ± 2.88 |
| 5 | 4-Hydroxybenzoic acid | 26.85 ± 3.35 |
| 6 | t-Ferulic acid | 20.7 ± 3.61 |
| 7 | Chlorogenic acid | 2.66 ± 1.56 |
| 8 | Rosmarinic acid | 1.58 ± 0.6 |
| 9 | t-Cinnamic acid | 1.25 ± 0.03 |
| 10 | Quercetin | 0.83 ± 0.01 |
| 11 | Naringenin | 0.78 ± 0.05 |
| 12 | o-Coumaric acid | 0.46 ± 0.01 |
| 13 | p-Coumaric acid | 0.18 ± 0.04 |
| 14 | Salicylic acid | 0.12 ± 0.04 |
| 15 | Resveratrol | 0.06 ± 0.01 |

Values represent mean ± standard deviation (n = 3). Results were obtained using HPLC-DAD analysis and expressed on a dry weight basis.

**Table S2.** Serum antioxidant enzyme levels across experimental groups.

|  | **Control** | **UV** | **Matcha** | **UV + Matcha** | ***P*-value** | **Effect size** |
| --- | --- | --- | --- | --- | --- | --- |
| **CAT** | 32.21 ± 7.82 | 33.15 ± 9.61 | 43.60 ± 9.86 | 46.51 ± 19.53 | 0.067 | 0.229 |
| **G6PD** | 2.54 ± 2.70 | 2.58 ± 0.99 | 3.38 ± 1.19 | 3.96 ± 1.75 | 0.369 | 0.108 |
| **GPx** | 654.95 (306.83–739.45) | 570.22 (544.29–739.72) | 730.94 (627.46–870.10) | 598.27 (180.92–822.52) | 0.522 | ≈ 0 (negligible) |
| **GR** | 96.15 (87.65–108.82) | 15.51 (12.12–17.18) | 24.10 (19.55–30.70) | 21.02 (17.93–31.66) | < 0.001 | 0.589 |
| **SOD** | 0.53 (0.52–0.64) | 0.56 (0.50–2.34) | 3.96 (2.93–4.69) | 4.07 (3.33–4.53) | < 0.001 | 0.597 |

UV, ultraviolet exposure; CAT, catalase; G6PD, glucose-6-phosphate dehydrogenase; GPx, glutathione peroxidase; GR, glutathione reductase; SOD, superoxide dismutase

Data are presented as mean ± SD or median (IQR), depending on data distribution. Group comparisons were performed using one-way ANOVA or Kruskal–Wallis test, as appropriate. Effect sizes are reported as η² for ANOVA and ε² for Kruskal–Wallis test. Post-hoc pairwise comparisons (Dunn–Bonferroni test): GR: Control vs UV, p < 0.001; SOD: Control vs Matcha, p = 0.005; Control vs UV + Matcha, p = 0.011; UV vs Matcha, p = 0.015; UV vs UV + Matcha, p = 0.031.

**Table S3.** Histomorphometric measurements of epidermal and dermal skin layers across study groups.

|  | **Control** | **UV** | **Matcha** | **UV + Matcha** | ***P*-value** | **Effect size** | **Intrarater ICC** | **Interrater ICC** |
| --- | --- | --- | --- | --- | --- | --- | --- | --- |
| Epidermal thickness (EpT) | 22.54 ± 6.4 | 61.47 ± 17.22 | 28.70 ± 7.86 | 34.13 ± 16.88 | < 0.001 | 0.596 | 0.997 (0.993–0.998)  0.997 (0.994–0.999) | 0.998  (0.996–0.999) |
| Papillary dermis thickness (PdT) | 85.87 ± 20.84 | 84.96 ± 25.73 | 92.95 ± 18.93 | 75.68 ± 20.87 | 0.452 | 0.084 | 0.996 (0.991–0.998)  0.992 (0.983–0.996) | 0.996  (0.991–0.998) |
| Reticular dermis thickness (RdT) | 1159.68 ± 77.02 | 1020.86 ± 116.23 | 1167.28 ± 170.53 | 908.14 ± 155.14 | 0.001 | 0.420 | 0.994 (0.984–0.997)  0.996 (0.992–0.998) | 0.979  (0.949–0.990) |
| Dermal papilla  thickness (DpT) | 53.68 ± 13.56 | 31.14 ± 14.23 | 44.01 ± 15.12 | 53.83 ± 19.42 | 0.023 | 0.284 | 0.982 (0.958–0.992)  0.988 (0.976–0.994) | 0.987  (0.968–0.994) |
| Epidermal rete ridge thickness (ErT) | 74.09 ± 25.92 | 29.10 ± 13.00 | 61.94 ± 17.12 | 88.07 ± 27.18 | < 0.001 | 0.537 | 0.992 (0.982–0.996)  0.994 (0.988–0.997) | 0.995  (0.991–0.998) |
| Interdigitation index (IDI) | 1.63 (1.60–1.69) | 1.07 (1.04–1.15) | 1.80 (1.74–2.02) | 1.38 (1.29–1.44) | < 0.001 | 0.884 | 0.990 (0.980–0.995)  0.996 (0.993–0.998) | 0.996  (0.991–0.998) |
| Granular cell density (GCD) | 46.65 ± 7.30 | 64.16 ± 21.63 | 52.03 ± 23.36 | 69.02 ± 17.27 | 0.023 | 0.213 | 0.994 (0.988–0.997)  0.996 (0.991–0.998) | 0.995  (0.991–0.998) |
| Spinous cell density | 111.19 (100.46–117.96) | 81.07 (71.29–187.39) | 145.10 (134.40–162.80) | 134.22 (99.57–178.40) | 0.133 | 0.093 | 0.999 (0.997–0.999)  0.998 (0.996–0.999) | 0.998  (0.997–0.999) |
| Basal cell density | 140.18 ± 24.75 | 106.06 ± 59.14 | 171.13 ± 48.52 | 178.49 ± 87.24 | 0.133 | 0.210 | 0.998 (0.997–0.999)  0.999 (0.998–0.999) | 0.998  (0.997–0.999) |

UV, ultraviolet exposure; ICC, intraclass correlation coefficient. Data are presented as mean ± SD or median (IQR), depending on data distribution. Group comparisons were performed using one-way ANOVA or Kruskal–Wallis test, as appropriate. Effect sizes are reported as η² for ANOVA and ε² for Kruskal–Wallis test. Post-hoc pairwise comparisons:

EpT (Games–Howell test): Control vs UV, p = 0.001; UV vs Matcha, p = 0.003; UV vs UV + Matcha, p = 0.029.

RdT (Tukey test): Control vs UV + Matcha, p = 0.004; Matcha vs UV + Matcha, p = 0.003.

DpT (Tukey test): Control vs UV, p = 0.037; UV vs UV + Matcha, p = 0.036.

ErT (Tukey test): Control vs UV, p = 0.001; UV vs Matcha, p = 0.025; UV vs UV + Matcha, p < 0.001.

IDI (Dunn–Bonferroni test): Control vs UV, p = 0.002; UV vs Matcha, p < 0.001; Matcha vs UV + Matcha, p = 0.009.

GCD (Games–Howell test): Control vs UV + Matcha, p = 0.032.

**Table S4.** Histological assessment of collagen and elastic fiber content based on Masson’s trichrome, PSR, and EVG staining.

|  | **Control** | **UV** | **Matcha** | **UV + Matcha** | ***P*-value** | **Effect size** | **Intrarater CC** | **Interrater ICC** |
| --- | --- | --- | --- | --- | --- | --- | --- | --- |
| Masson’s trichrome-stained area (%) | 26.75 ± 4.20 | 37.38 ± 4.41 | 27.13 ± 4.02 | 34.00 ± 3.67 | < 0.001 | 0.586 | 0.901 (0.460–0.967)  0.896 (0.776–0.950) | 0.884  (0.491–0.958) |
| PSR (Total Collagen, %) | 41.63 ± 4.05 | 33.36 ± 7.86 | 35.51 ± 4.11 | 29.03 ± 5.73 | 0.001 | 0.424 | 0.908 (0.812–0.955)  0.938 (0.863–0.971) | 0.935  (0.866–0.968) |
| PSR (Type I collagen, %) | 32.72 ± 4.58 | 17.26 ± 4.95 | 28.08 ± 4.35 | 19.68 ± 4.82 | < 0.001 | 0.671 | 0.925 (0.848–0.963)  0.925 (0.847–0.964) | 0.940  (0.877–0.971) |
| PSR (Type III collagen, %) | 7.56 (5.44–8.37) | 13.78 (11.47–17.02) | 5.03 (4.22–6.30) | 7.35 (6.14–11.21) | 0.001 | 0.506 | 0.867 (0.727–0.935)  0.885 (0.766–0.944) | 0.849  (0.687–0.927) |
| PSR (Type I/III collagen) | 4.10 (3.48–9.24) | 1.19 (0.95–1.39) | 6.14 (4.45–6.98) | 2.11 (1.91–2.84) | < 0.001 | 0.657 | 0.892 (0.780–0.947)  0.899 (0.793–0.951) | 0.914  (0.823–0.958) |
| EVG-stained area (%) | 98.19 ± 0.42 | 97.03 ± 1.65 | 96.90 ± 0.90 | 96.28 ± 1.32 | 0.002 | 0.297 | 0.776 (0.539–0.891)  0.772 (0.505–0.892) | 0.811  (0.558–0.914) |
| EVG (circ 0-1) | 0.961 ± 0.013 | 0.961 ± 0.008 | 0.961 ± 0.005 | 0.963 ± 0.008 | 0.938 | ≈ 0 (negligible) | 0.930 (0.857–0.966)  0.956 (0.911–0.979) | 0.953  (0.904–0.977) |
| EVG (solidity 0-1) | 0.962 ± 0.012 | 0.964 ± 0.012 | 0.964 ± 0.007 | 0.962 ± 0.008 | 0.970 | ≈ 0 (negligible) | 0.943 (0.883–0.972)  0.959 (0.916–0.980) | 0.950  (0.896–0.976) |

UV, ultraviolet exposure; PCR, Picrosirius Red staining; EVG, Elastic van Gieson staining; ICC, intraclass correlation coefficient. Data are presented as mean ± SD or median (IQR), depending on data distribution. Group comparisons were performed using one-way ANOVA or Kruskal–Wallis test, as appropriate. Effect sizes are reported as η² for ANOVA and ε² for Kruskal–Wallis test. Post-hoc pairwise comparisons:

Trichrome (%) (Tukey test): Control vs UV, p < 0.001; Control vs UV + Matcha, p = 0.007; UV vs Matcha, p < 0.001; Matcha vs UV + Matcha, p = 0.011.

PSR-Total collagen (%) (Tukey test): Control vs UV, p = 0.032; Control vs UV + Matcha, p = 0.001.

PSR-Type I collagen (%) (Tukey test): Control vs UV, p < 0.001; Control vs UV + Matcha, p < 0.001; UV vs Matcha, p < 0.001; Matcha vs UV + Matcha, p = 0.006.

PSR-Type III collagen (%) (Dunn–Bonferroni test): Control vs UV, p = 0.016; UV vs Matcha p < 0.001.

PSR-Type I/III ratio (Dunn–Bonferroni test): Control vs UV, p = 0.001; UV vs Matcha, p < 0.001.

EVG-% area (Tukey test): Control vs Matcha, p = 0.018; Control vs UV + Matcha, p = 0.015.

**Table S5.** Macroscopic skin characteristics and surface texture parameters across experimental groups.

|  | **Control** | **UV** | **Matcha** | **UV + Matcha** | ***P*-value** | **Effect size** | **Intrarater ICC** | **Interrater ICC** |
| --- | --- | --- | --- | --- | --- | --- | --- | --- |
| L* | 72.63  (71.48–73.18) | 65.62  (64.74–66.22) | 70.43  (70.24–70.91) | 68.28  (67.85–68.89) | < 0.001 | 0.872 | 0.768 (0.524–0.887)  0.757 (0.504–0.882) | 0.777  (0.499–0.896) |
| a* | 3.72  (1.26–4.24) | 5.48  (5.27–6.72) | 3.89  (3.79–4.25) | 5.17  (4.72–5.28) | < 0.001 | 0.734 | 0.885 (0.747–0.946)  0.841 (0.675–0.922) | 0.862  (0.718–0.932) |
| b* | -2.81 ± 1.50 | -2.42 ± 2.35 | -2.77 ± 0.34 | -3.15 ± 0.67 | 0.566 | 0.036 | 0.912 (0.821–0.957)  0.896 (0.788–0.949) | 0.905  (0.807–0.954) |
| FFT Roughness index | 21.88 ± 1.46 | 36.75 ± 4.53 | 23.38 ± 1.51 | 32.88 ± 4.39 | 0.001 | 0.803 | 0.978 (0.956–0.989)  0.979 (0.958–0.990) | 0.977  (0.952–0.989) |
| Local variance index | 48.50 ± 5.76 | 75.88 ± 5.28 | 49.88 ± 1.46 | 67.38 ± 5.78 | < 0.001 | 0.865 | 0.990 (0.980–0.995)  0.978 (0.956–0.989) | 0.984  (0.967–0.992) |
| Edge mean | 29.63 ± 2.97 | 38.50 ± 1.77 | 29.13 ± 2.03 | 35.75 ± 1.49 | < 0.001 | 0.800 | 0.875 (0.746–0.939)  0.919 (0.826–0.961) | 0.873  (0.725–0.940) |
| Wrinkle (Total, %) | 3.40 ± 0.20 | 7.44 ± 0.35 | 2.59 ± 0.08 | 7.03 ± 0.22 | < 0.001 | 0.990 | 0.957 (0.913–0.979)  0.962 (0.923–0.982) | 0.966  (0.930–0.983) |
| Wrinkle (Fine, %) | 1.93 ± 0.14 | 6.04 ± 0.28 | 2.37 ± 0.74 | 5.43 ± 0.17 | < 0.001 | 0.991 | 0.911 (0.817–0.957)  0.865 (0.723–0.934) | 0.903  (0.802–0.952) |
| Wrinkle (Coarse, %) | 1.53 ± 0.07 | 1.42 ± 0.10 | 0.36 ± 0.08 | 1.57 ± 0.07 | < 0.001 | 0.977 | 0.873 (0.682–0.943)  0.943 (0.873–0.974) | 0.886  (0.716–0.949) |

L*, lightness; a*, redness (red–green color axis); b*, yellow–blue color axis; FFT, Fast Fourier Transform; ICC, intraclass correlation coefficient. Data are presented as mean ± SD or median (IQR), depending on data distribution. Group comparisons were performed using one-way ANOVA or Kruskal–Wallis test, as appropriate. Effect sizes are reported as η² for ANOVA and ε² for Kruskal–Wallis test. Post-hoc pairwise comparisons:

L* (Dunn–Bonferroni test): Control vs UV, p < 0.001; Control vs UV + Matcha, p = 0.011; UV vs Matcha, p = 0.001.

a* (Dunn–Bonferroni test): Control vs UV, p < 0.001; Control vs UV + Matcha, p = 0.029; UV vs Matcha, p = 0.001.

FFT Roughness Index (Games–Howell test): Control vs UV, p < 0.001; Control vs UV + Matcha, p = 0.001; UV vs Matcha, p < 0.001; Matcha vs UV + Matcha, p = 0.001.

Local Variance Index (Games–Howell test): Control vs UV, p < 0.001; Control vs UV + Matcha, p < 0.001; UV vs Matcha, p < 0.001; UV vs UV + Matcha, p = 0.037; Matcha vs UV + Matcha, p < 0.001.

Edge Mean (Tukey test): Control vs UV, p < 0.001; Control vs UV + Matcha, p < 0.001; UV vs Matcha, p < 0.001; Matcha vs UV + Matcha, p < 0.001.

Wrinkle (total, %) (Games–Howell test): Control vs UV, p < 0.001; Control vs Matcha, p < 0.001; Control vs UV + Matcha, p < 0.001; UV vs Matcha, p < 0.001; Matcha vs UV + Matcha, p < 0.001.

Wrinkle (fine, %) (Games–Howell test): Control vs UV, p < 0.001; Control vs Matcha, p < 0.001; Control vs UV + Matcha, p < 0.001; UV vs Matcha, p < 0.001; UV vs UV + Matcha, p = 0.001; Matcha vs UV + Matcha, p < 0.001.

Wrinkle (coarse, %) (Tukey test): Control vs UV, p = 0.037; Control vs Matcha, p < 0.001; UV vs Matcha, p < 0.001; UV vs UV + Matcha, p = 0.005; Matcha vs UV + Matcha, p < 0.001.

**Table S6.** Correlations between serum antioxidant enzymes and histological, macroscopic, and extracellular matrix parameters.

| **Correlations** | **r_s_** | ***P*-value** |
| --- | --- | --- |
| SOD–Spinous cell density | 0.555 | 0.001 |
| SOD–Basal cell density | 0.356 | 0.049 |
| SOD–EVG-stained area (%) | - 0.419 | 0.019 |
| GR–Epidermal thickness | - 0.592 | < 0.001 |
| GR–Epidermal rete ridge thickness | 0.403 | 0.025 |
| GR–IDI | 0.413 | 0.021 |
| GR–Masson’s trichrome (%) | - 0.370 | 0.040 |
| GR–EVG (IntDens) | 0.465 | 0.008 |
| GR–PSR (Total collagen, %) | 0.540 | 0.002 |
| GR–PSR (Type I collagen, %) | 0.677 | < 0.001 |
| GR–PSR (Type III collagen, %) | - 0.426 | 0.017 |
| GR–PSR (Type I/III collagen) | 0.547 | 0.001 |
| GR–L^*^ | 0.649 | < 0.001 |
| GR–a^*^ | 0.578 | 0.001 |
| GR–FFT Roughness index | - 0.612 | < 0.001 |
| GR–Local Variance index | - 0.536 | 0.002 |
| GR–Edge mean | - 0.542 | 0.002 |
| GR–Wrinkle (Total, %) | - 0.454 | 0.010 |
| GR–Wrinkle (Fine, %) | - 0.787 | < 0.001 |
| CAT–EVG-stained area (%) | - 0.361 | 0.046 |
| G6PD–PCR (Total collagen %) | - 0.358 | 0.048 |
| GPx–EVG (circ) | 0.362 | 0.046 |
| CAT–G6PD | 0.496 | 0.005 |
| CAT–SOD | - 0.459 | 0.009 |

SOD, superoxide dismutase; r_s_, Spearman correlation coefficient; GR, glutathione reductase; IDI, interdigitation index; EVG, Elastic van Gieson staining; PSR, Picrosirius Red staining; GPx, glutathione peroxidase; circ, circularity; CAT, catalase; G6PD, glucose-6-phosphate dehydrogenase.

**Table S7**. Correlations between macroscopic surface texture indices and histological skin parameters.

| **Correlations** | **r_s_** | ***P*-value** |
| --- | --- | --- |
| FFT Roughness index–Epidermal thickness | 0.522 | 0.002 |
| FFT Roughness index–Reticular dermis thickness | - 0.491 | 0.004 |
| FFT Roughness index–Epidermal rete ridge thickness | - 0.390 | 0.027 |
| FFT Roughness index–IDI | - 0.788 | < 0.001 |
| FFT Roughness index–Granular cell density | 0.447 | 0.010 |
| FFT Roughness index–Trichrome (%) | 0.690 | < 0.001 |
| FFT Roughness index–EVG-stained area (%) | - 0.379 | 0.032 |
| FFT Roughness index–PSR (Total collagen, %) | - 0.551 | 0.001 |
| FFT Roughness index–PSR (Type I collagen, %) | - 0.787 | < 0.001 |
| FFT Roughness index–PSR (Type III collagen, %) | 0.642 | < 0.001 |
| FFT Roughness index–PSR (Type I/III collagen) | - 0.787 | < 0.001 |
| IDI–Epidermal thickness | - 0.503 | 0.003 |
| Local variance index–Epidermal thickness | 0.519 | 0.002 |
| Local variance index–Reticular dermis thickness | - 0.557 | 0.001 |
| Local variance index–Epidermal rete ridge thickness | - 0.387 | 0.029 |
| Local variance index–IDI | - 0.838 | < 0.001 |
| Local variance index–Granular cell density | 0.411 | 0.020 |
| Local variance index–Trichrome (%) | 0.644 | < 0.001 |
| Local variance index–PSR (Total collagen, %) | - 0.377 | 0.034 |
| Local variance index–PSR (Type I collagen, %) | - 0.658 | < 0.001 |
| Local variance index–PSR (Type III collagen, %) | 0.659 | < 0.001 |
| Local variance index–PSR (Type I/III collagen) | - 0.771 | < 0.001 |
| Edge mean–Epidermal thickness | 0.535 | 0.002 |
| Edge mean–Reticular dermis thickness | - 0.523 | 0.002 |
| Edge mean–IDI | - 0.840 | < 0.001 |
| Edge mean–Granular cell density | 0.465 | 0.007 |
| Edge mean–Masson’s trichrome (%) | 0.700 | < 0.001 |
| Edge mean–PSR (Total collagen, %) | - 0.416 | 0.018 |
| Edge mean–PSR (Type I collagen, %) | - 0.705 | < 0.001 |
| Edge mean–PSR (Type III collagen, %) | 0.674 | < 0.001 |
| Edge mean–PSR (Type I/III collagen) | - 0.799 | < 0.001 |
| Wrinkle (Total, %)–Epidermal thickness | 0.450 | 0.010 |
| Wrinkle (Total, %)–Reticular dermis thickness | - 0.501 | 0.003 |
| Wrinkle (Total, %)–IDI | - 0.838 | < 0.001 |
| Wrinkle (Total, %)–Trichrome (%) | 0.623 | < 0.001 |
| Wrinkle (Total, %)–PSR (Type I collagen, %) | - 0.613 | < 0.001 |
| Wrinkle (Total, %)–PSR (Type III collagen, %) | 0.642 | < 0.001 |
| Wrinkle (Total, %)–PSR (Type I/III collagen) | - 0.700 | < 0.001 |
| Wrinkle (Fine, %)–Epidermal thickness | 0.614 | < 0.001 |
| Wrinkle (Fine, %)–Reticular dermis thickness | - 0.503 | 0.003 |
| Wrinkle (Fine, %)–Dermal papilla thickness | - 0.359 | 0.044 |
| Wrinkle (Fine, %)–Epidermal rete ridge thickness | - 0.425 | 0.015 |
| Wrinkle (Fine, %)–IDI | - 0.744 | < 0.001 |
| Wrinkle (Fine, %)–Masson’s trichrome (%) | 0.701 | < 0.001 |
| Wrinkle (Fine, %)–EVG-stained area (%) | - 0.431 | 0.014 |
| Wrinkle (Fine, %)–PSR (Total collagen, %) | - 0.531 | 0.002 |
| Wrinkle (Fine, %)–PSR (Type I collagen, %) | - 0.764 | < 0.001 |
| Wrinkle (Fine, %)–PSR (Type III collagen, %) | 0.565 | 0.001 |
| Wrinkle (Fine, %)–PSR (Type I/III collagen) | - 0.704 | < 0.001 |

r_s_, Spearman correlation coefficient; FFT, Fast Fourier Transform; IDI, interdigitation index; PSR, Picrosirius Red staining; EVG, Elastic van Gieson staining

**Table S8.** Correlations between collagen composition, elastic fiber parameters, and histomorphometric features.

| **Correlations** | **r_s_** | ***P*-value** |
| --- | --- | --- |
| PSR (Total collagen, %)–Granular cell density | - 0.364 | 0.041 |
| PSR (Total collagen, %)–Reticular dermis thickness | 0.468 | 0.007 |
| PSR (Type I collagen, %)–Granular cell density | - 0.389 | 0.028 |
| PSR (Type I collagen, %)– Reticular dermis thickness | 0.496 | 0.004 |
| PSR (Type III collagen, %)–Epidermal rete ridge thickness | - 0.528 | 0.002 |
| PSR (Type I/III collagen)–Epidermal rete ridge thickness | 0.516 | 0.002 |
| PSR (Type I/III collagen)–Reticular dermis thickness | 0.399 | 0.024 |
| EVG-stained area (%)–Reticular dermis thickness | 0.464 | 0.008 |
| EVG-stained area (%)–Granular cell density | - 0.376 | 0.034 |
| EVG-stained area (%)–Basal cell density | - 0.352 | 0.048 |

r_s_, Spearman correlation coefficient; PSR, Picrosirius Red staining; EVG, Elastic van Gieson staining
