# Supplementary material for: Oral whole-leaf matcha partially attenuates UV-induced dermoepidermal disruption and collagen phenotype alterations in a rat model of repeated photoaging
Source: Front Med (Lausanne). 2026 Jun 10;13:1813454. doi: 10.3389/fmed.2026.1813454 (PMC13290547; doi:10.3389/fmed.2026.1813454)
Supplement: Supplementary file 3 [file Table_2.docx]

**Supplementary Methods.** Detailed stepwise image analysis procedures performed using FIJI/ImageJ software.

**1) Images of H&E stained sections:**

**Parameters:** Epidermal thickness (EpT), papillary dermis thickness (PdT), reticular dermis thickness (RdT), dermal papilla thickness (DpT), epidermal rete ridge thickness (ErT), interdigitation index (IDI), and cellularity in epidermal layers.

**General setup (applies to all measurements)**

1. The image scale was calibrated using *Analyze > Set Scale* (reference micrometer).
2. Magnification/scale settings were kept constant for all sections.
3. For each section, the region of interest (ROI) was defined to include the target structure and exclude artifacts (folds, tears, staining deposits).
4. For each animal, five independent sections were analyzed. On each section, five randomly selected non-overlapping fields were selected, and one measurement per parameter was obtained from each field, yielding 25 measurements (5 slides × 5 fields) per parameter per animal. The mean value per animal was used for statistical analysis.

**1.1. Epidermal Thickness (μm)**

1. Measurements were taken along a perpendicular axis from the basement membrane (below stratum basale) toward the outermost stratum corneum using *Straight Line*.
2. Analyze > Measure was used to measure the distance.
3. The length was recorded in μm.
4. One epidermal thickness measurement was obtained from each selected field. A total of 25 measurements per animal were obtained (5 sections × 5 fields), and the mean value per animal was recorded as the epidermal thickness.

**1.2. Papillary Dermis Thickness (PdT, μm)**

1. The lower border of the epidermal rete ridge (papillary crest base) and the transition to reticular dermis (identified based on morphological transition to thicker and more densely packed collagen bundles) were determined.
2. The path between these two borders was traced using *Freehand Line* tool.
3. *Analyze > Measure* was used to measure the distance.
4. Length was recorded in μm.
5. One measurement was obtained from each selected field. A total of 25 measurements per animal were obtained (5 sections × 5 fields), and the mean value per animal was recorded as the papillary dermis thickness.

**1.3. Reticular Dermis Thickness (RdT, μm)**

1. The lower border of the papillary dermis and the junction with subcutaneous adipose tissue were identified.
2. The path along the dermal contour was traced using the *Segmented Line* (or *Freehand Line*, when applicable) tool.
3. *Analyze > Measure* was used to measure the distance.
4. The length was recorded in μm.
5. One measurement was obtained from each selected field. A total of 25 measurements per animal were obtained (5 sections × 5 fields), and the mean value per animal was recorded as the reticular dermis thickness.

**1.4. Dermal Papillae Thickness (DpT, μm)**

1. Individual dermal papillae (dermal projections toward the epidermis) were identified.
2. A line was drawn from the papilla base (dermal surface) to the papilla tip (adjacent to the basement membrane) using *Freehand Line* tool.
3. *Analyze > Measure* was used to measure the distance.
4. The length was recorded in μm.
5. One measurement was obtained from each selected field. A total of 25 measurements per animal were obtained (5 sections × 5 fields), and the mean value per animal was recorded as the dermal papilla thickness.

**1.5. Epidermal Rete Ridge Thickness (ErT, μm)**

1. Individual rete ridges (downward epidermal projections) were identified.
2. A line was drawn from the ridge tip to the deepest point along the basement membrane using the *Freehand Line* tool.
3. *Analyze > Measure* was used to measure the distance.
4. The length was recorded in μm.
5. One measurement was obtained from each selected field. A total of 25 measurements per animal were obtained (5 sections × 5 fields), and the mean value per animal was recorded as the epidermal rete ridge thickness.

**1.6. Interdigitation Index (IDI)**

1. The image scale was calibrated using *Analyze > Set Scale* with the reference micrometer, and the measurement unit was set to μm.
2. The epidermal–dermal junction was traced along its natural undulating contour using the *Freehand Line* tool, and the length was measured (*Analyze > Measure*).
3. A straight reference line crossing the same region horizontally was drawn using the *Straight Line* tool, and its length was measured (*Analyze > Measure*).
4. The IDI was calculated as the ratio of the contour length to the straight reference length.

An IDI value greater than 1 indicates increased epidermal–dermal interface complexity.

**1.7. Epidermal Cell Density (Granular, Spinous and Basal Cell Densities) (cells/mm²)**

1. The image scale was calibrated using *Analyze > Set Scale* (reference μm).
2. Within the epidermis, a ROI was defined using the *Polygon Selection* tool.
3. The ROI area was measured with *Analyze > Measure* and recorded in µm².
4. Epidermal cells were manually counted using the *Plugins > Analyze > Cell Counter* tool. Only nuclei with clearly visible boundaries were included in the counts.
   - Granular, spinous, and basal layer cell nuclei within the selected ROI were marked separately using different counter types (Type 1, Type 2, Type 3) in the Cell Counter plugin
   - The total cell counts for each epidermal layer were displayed in the Cell Counter window.
5. Cell densities for the granular, spinous, and basal layers were calculated as:

Cell density (cells/mm²) = (Cell count / ROI area [µm²]) × 1,000,000

**2) Masson trichrome–stained sections**

**Parameters:** Dermal collagen area fraction (%)

1. The image scale was calibrated using *Analyze > Set Scale*.
2. The image was separated into individual RGB channels using *Image > Color > Split Channels*.
3. The blue channel, corresponding to collagen staining, was selected for analysis.
4. Thresholding was applied (*Image > Adjust > Threshold*) and sliders were adjusted to isolate the collagen-stained regions; a binary mask was generated using *Apply*. A consistent threshold range was applied across all images.
5. Measurement parameters (area, area fraction, limit to threshold) were selected in *Analyze > Set Measurements*.
6. A ROI encompassing the full dermal compartment while excluding epidermis and subcutaneous tissue was defined using the *Polygon Selection* tool.
7. Collagen content was quantified using *Analyze > Measure*, and the area fraction (%) within the ROI was recorded as the collagen %.

All measurements were performed on calibrated images under identical thresholding and display settings to ensure consistency across samples.

**3)** **Elastic van Gielson (EVG)–stained sections**

**Parameters:** Elastin fiber area fraction (%), integrated density, circularity, and solidity.

1. The image scale was calibrated using *Analyze > Set Scale*.
2. Elastin fibers were segmented using the *Plugins > Segmentation >* *Trainable Weka Segmentation*. A pretrained classifier was loaded, including classes such as “Elastin” and “Background,” using default feature settings.
3. A segmentation output image was generated using *Create Result*.
4. The elastin component was isolated using *Image > Adjust > Threshold*, and a binary mask was created with *Apply* using the same threshold parameters for all samples.
5. Measurement parameters (area, area fraction, shape descriptors) were selected in *Analyze > Set Measurements*.
6. A ROI encompassing the full dermal compartment while excluding epidermis and subcutaneous tissue was defined using the Polygon Selection tool.
7. Elastin fibers were quantified using *Analyze > Analyze Particles*, and the following metrics were recorded: area fraction (%), circularity, solidity.

All measurements were performed on calibrated images under identical thresholding and display settings to ensure consistency across samples.

**4)** **Picrosirius red (PCR)–stained sections**

**Parameters:** Total collagen, type I collagen, and type III collagen area fraction (%)

**A) Type I and Type III Collagen Analysis**

1. The image scale was calibrated using *Analyze > Set Scale*.
2. Images acquired under polarized light microscopy were separated into RGB channels using *Image > Color > Split Channels*.
3. The red channel was used to quantify predominantly type I collagen fibers (red/orange birefringence), and the green channel was used to quantify predominantly type III collagen fibers (green birefringence).
4. Thresholding was performed using *Image > Adjust > Threshold*, with dark background enabled. Threshold values were determined on representative images and then applied uniformly across all samples.
5. A binary mask was created using *Process > Binary > Make Binary*.
6. A dermal ROI was defined using the Polygon Selection tool and added to the ROI Manager.
7. Area fraction was selected in *Analyze > Set Measurements*. Collagen content was expressed as area fraction (%) within the dermal ROI. Other particle-based metrics (mean gray value, integrated density, circularity, solidity, count, average size) were not used, as all analyses were performed on binary masks.
8. Collagen fibers within the ROI were quantified using *Analyze > Analyze Particles*, and the area fraction (%) was recorded as the collagen %.

**B) Total Collagen Analysis**

1. The image scale was calibrated using *Analyze > Set Scale*.
2. Images were converted to HSB color space using *Image > Type > HSB Stack*.
3. Total collagen was isolated using *Image > Adjust > Color Threshold* (mode: HSB), with manually selected threshold values kept constant for all samples.
4. A binary mask was created using *Select > Create Mask*.
5. A dermal ROI was defined using the *Polygon Selection* tool.
6. Measurement settings were selected as above, with “limit to threshold” enabled.
7. Total collagen area fraction (%) within the ROI was quantified using *Analyze > Analyze Particles*.

All measurements were performed on calibrated images under identical thresholding and display settings to ensure consistency across samples.

**5. Macroscopic Images**

**Parameters:** Surface texture/coarseness, pigmentation, and wrinkle-related indices

**5.1. Texture/Coarseness Analysis**

1. The image scale was calibrated using the *Set Scale* according to the embedded reference ruler.
2. A ROI was manually defined for each sample.
3. Uneven background illumination was corrected using *Process > Subtract Background* (rolling-ball radius: 100 px).
4. Local contrast was enhanced using *Process > Enhance Local Contrast (CLAHE;* block size: 127; slope: 3.0).
5. Each image was converted to 8-bit (*Image > Type > 8-bit*).
6. A bandpass filter was applied (*Process > FFT > Bandpass Filter)* with large structures down to 40 px and small structures up to 2 px, (“Autoscale after filtering” enabled; “Saturate large pixels” disabled).
7. Surface roughness (*roughness index)* was quantified (*Analyze > Measure*) as the standard deviation of pixel intensities within the ROI.
8. *The local variance index* was determined using *Process > Filters > Variance* (Radius: 3 px), and the mean value was recorded.
9. *Edge intensity* was measured using *Process > Find Edges*, and the mean pixel intensity within the ROI was recorded (*Analyze > Measure*).

All parameters (filter sizes, radii, and contrast settings) were kept constant across all images to ensure consistency and reproducibility.

**5.2. Wrinkle Analysis**

1. The image scale was calibrated using *Set Scale* according to the embedded reference ruler.
2. A ROI was manually defined for each sample.
3. Uneven background illumination was corrected using *Process > Subtract Background* (rolling-ball radius: 100 px).
4. Local contrast was enhanced using *Process > Enhance Local Contrast (CLAHE;* block size: 127; slope: 3.0).
5. Each image was converted to 8-bit (*Image > Type > 8-bit*).
6. A bandpass filter was applied (*Process > FFT > Bandpass Filter*) with large structures down to 40 px and small structures up to 2 px (“Autoscale after filtering” enabled; “Saturate large pixels” disabled).
7. Thresholding was adjusted (*Image > Adjust > Threshold*) to separately identify fine and coarse wrinkle lines by setting low and high threshold levels, respectively.
8. Total, fine, and coarse wrinkle area fractions (%) within the ROI were quantified using *Analyze > Measure.*

All thresholding and filtering parameters were kept constant across all images to ensure consistency and reproducibility.

**5.3. Pigmentation Analysis**

1. The image scale was calibrated using *Set Scale* according to the embedded reference ruler.
2. A ROI was manually defined for each sample.
3. Each image was split into separate color channels using *Image > Color > Split Channels*, producing red (R), green (G), and blue (B) channel images. RGB images were analyzed without compression artifacts or automatic color enhancement.
4. For each channel, *Analyze > Measure* was used to record the mean pixel intensity within the defined ROI.
5. The resulting RGB intensity data were exported to Python (Google Colab) and converted to the CIE L*a*b* color space using the *skimage.color.rgb2lab* function, assuming the standard sRGB color space.
6. Mean L*, a*, and b* values were calculated for each sample to quantify pigmentation brightness (L*) and chromaticity (a* and b*).

All image processing and color conversion parameters were kept constant across all samples to ensure consistency and reproducibility.
